# Application of Methods to Assess Animal Welfare and Suffering Caused by Infectious Diseases in Cattle and Swine Populations

**DOI:** 10.3390/ani11113017

**Published:** 2021-10-20

**Authors:** Søren Saxmose Nielsen, Hans Houe, Matthew Denwood, Liza Rosenbaum Nielsen, Björn Forkman, Nina Dam Otten, Jens Frederik Agger

**Affiliations:** Section for Animal Welfare and Disease Control, Department of Veterinary and Animal Sciences, University of Copenhagen, 1870 Frederiksberg C, Denmark; houe@sund.ku.dk (H.H.); md@sund.ku.dk (M.D.); liza@sund.ku.dk (L.R.N.); bjf@sund.ku.dk (B.F.); nio@sund.ku.dk (N.D.O.); jfa@sund.ku.dk (J.F.A.)

**Keywords:** animal welfare, Aujeszky´s disease, bovine viral diarrhoea, infectious bovine rhinotracheitis, *Mycobacterium avium* subsp. *paratuberculosis*, porcine respiratory and reproductive syndrome

## Abstract

**Simple Summary:**

Infectious disease control in livestock is often motivated by food safety concerns and economic impact. However, most diseases also affect animal welfare. We established an approach to quantify the welfare effect of infectious diseases in cattle (three diseases) and pigs (two diseases). A “suffering score” was established based on the aggregation of severity, duration, and frequency of clinical entities of the diseases. The resulting suffering scores were then used to compare the welfare impact of the different diseases and for comparison to other common welfare hazards. For example, the approach suggested that bovine viral diarrhoea and paratuberculosis are more severe for cattle than infectious bovine rhinotracheitis. In pigs, porcine reproductive and respiratory syndrome has a much bigger welfare impact than Aujeszky’s disease, assuming all diseases remain endemic.

**Abstract:**

Control of infectious diseases in livestock has often been motivated by food safety concerns and the economic impact on livestock production. However, diseases may also affect animal welfare. We present an approach to quantify the effect of five infectious diseases on animal welfare in cattle (three diseases) and pigs (two diseases). We grouped clinical manifestations that often occur together into lists of clinical entities for each disease based on literature reviews, and subsequently estimated “suffering scores” based on an aggregation of duration, frequency, and severity. The duration and severity were based on literature reviews and expert knowledge elicitation, while frequency was based mainly on estimates from the literature. The resulting suffering scores were compared to scores from common welfare hazards found under Danish conditions. Most notably, the suffering scores for cattle diseases were ranked as: bovine viral diarrhoea and infection with *Mycobacterium avium* subsp. *paratuberculosis* > infectious bovine rhinotracheitis, and for pigs as: porcine respiratory and reproductive syndrome > Aujeszky’s disease. The approach has limitations due to the limited data available in literature and uncertainties associated with expert knowledge, but it can provide decision makers with a tool to quantify the impact of infections on animal welfare given these uncertainties.

## 1. Introduction

Infectious diseases are common in livestock, where they may be controlled or eradicated due to their impact on food security, food safety, farm economy, and other types of societal impact. These motives have been the main drivers behind the organised control of many infectious diseases in livestock [1]. However, animal welfare is also a reason mentioned in the European Union Animal Health Law [2], a regulation that was adopted by the European Parliament in 2016 and implemented in April 2021. Signs of disease have been associated with animal welfare consequences in the individual, or at group level, and are often included in animal welfare protocols, especially those that focus on input variables (e.g., in Welfare Quality^®^ [3] and KTBL (Das Kuratorium für Technik und Bauwesen in der Landwirtschaft e.V.) [4]. However, since these protocols focus on assessing the welfare on farm, often by non-veterinarians, it is clinical signs that are included in the protocols, rather than the diseases. To our knowledge, the impact of livestock diseases on animal welfare has not been quantified systematically, neither at individual nor at population level, and no standard methods exist to allow for such animal welfare impact assessments.

Infectious diseases can affect animal welfare in multiple ways, e.g., reduced comfort of the individual due to the acute pathologies caused by the infectious agent resulting in clinical signs such as fever, weakness, and diarrhoea, or long-term effects where weight loss and general unthriftiness may place the animal in a lower ranking in an animal group. Reduced animal welfare may also result from lack of social interaction due to disease control measures imposed on the entire population, e.g., if calf and dam are separated shortly after calving to mitigate the risk of transmission from dam to calf.

The aim of this project was to develop and illustrate a new approach to assess the impact of infectious diseases on animal welfare in livestock. The five diseases are bovine virus diarrhoea (BVD), infectious bovine rhinotracheitis (IBR) and infection with *Mycobacterium avium* subsp. *paratuberculosis* (MAP) in dairy cattle, and Aujeszky’s disease and porcine respiratory and reproductive syndrome (PRRS) in pigs. These diseases were primarily selected due to the long-standing legal requirements to control these diseases in Denmark, but not necessarily in the European Union. Furthermore, the diseases are quite different and represent different areas of potential suffering.

The objectives of the study (exemplified with these five diseases) were to:

- develop a measurement scale for assessing the level of animal welfare and the impact of disease on animal welfare (pain and general discomfort);
- estimate the duration and severity scores for each disease and clinical entity based on expert knowledge elicitation (EKE);
- estimate animal suffering scores for common non-infectious welfare challenges (such as broken femur, lack of access to water, separation of dam and offspring) in pig and cattle production for comparison and perspective;
- combine duration, severity, and frequency into an aggregated suffering score.

## 2. Materials and Methods

### 2.1. Overview

The work was based on a summary of the literature on disease manifestations of each of the five diseases. These were grouped into different “clinical entities” (i.e., disease stages with clinical signs that typically occur concurrently for the disease in question) and a summary of the occurrence and duration of the diseases based on literature reviews and EKEs. The assessment using EKEs was performed based on a derivative of the Delphi method [5], which has previously been used for infectious diseases and animal welfare in connection with the Animal Health Law [6,7,8,9]. The approach consists of a two-step individual assessment followed by collective behavioural aggregation [5].

The term “clinical entity” is used to cover disease manifestations with multiple clinical signs present at the same time, e.g., “diarrhoea and emaciation” or “nasal discharge and pneumonia”. Different clinical entities of the same disease were assumed to be mutually exclusive.

### 2.2. Literature Summary of Diseases and Their Clinical Entities

Literature reviews for BVD virus, IBR virus, and MAP infections in dairy cattle and Aujeszky’s disease and PRRS in pigs have previously been carried out (see [10] and Supplementary Materials, where extensive literature searches are provided), giving information on clinical entities and the related typical signs as well as their duration and frequency. The clinical entities and associated clinical signs for relevant age groups are listed in Table 1. An additional ten welfare hazards (five for cattle and five for pigs) not related to the infectious diseases were also selected for assessment for comparative purposes (Table 2).

### 2.3. Animal Welfare Scoring and Assessment

#### 2.3.1. Expert Knowledge Elicitation of Severity and Duration

Quantification of animal welfare is a challenge, but expert judgement is commonly used in food and feed safety risk assessments, and has also been used in animal welfare judgements [7,11]. Expert elicitation methods include the Sheffield method, where behavioural aggregation is obtained through face-to-face discussions between experts, and the Cooke method, which employs mathematical aggregation. The Delphi method lies in between these options, as it allows some restricted interaction between experts [5]. The step-wise EKE was carried out as listed in the Box 1.

Box 1 **Stepwise expert knowledge elicitation.**

An EKE with subsequent behavioural aggregation among the experts was carried out according to the following steps. Each of the eight experts (B.F., H.H., J.F.A., L.R.N., M.D., N.D.O., S.S.N., and a final year veterinary student) were asked to create a score for each clinical entity based on the following instruction: Imagine that ”severity” is described on a scale of 0 to 10, where 0 is “no noticeable effect” and 10 is “unbearable pain or other negative effect” for the animal. “Mastitis” in cattle was set as a reference value of 5.

The question to be addressed for each clinical entity was then:

Imagine that 1000 animals are affected with this clinical entity for a period with the duration described by duration_q1, duration_q2 and duration_q3, i.e., the interquartile range (q1–q3) and the median (q2). What would be the distribution of the combined severity of the typical signs during this period? Please describe the severity distribution with the 2.5 percentile, the mode (most likely), and the 97.5 percentile.

This first step was carried out for each disease and constituted the individual assessment. Step 2 was a group discussion, which occurred in virtual group meetings (due to restrictions imposed due to the national management of SARS-CoV-2 circulation in Denmark in 2020). The participants saw the scores of the others and were asked to verbally rationalise their choices, ask questions to other participants about their motivation, and ultimately re-score if they felt they had not taken sufficient aspects into account during their initial individual scoring. It was stressed that all participants were entitled to keep their original score. Finally, a distribution of scores for each clinical entity was included.

A similar approach was taken for the selected non-infectious welfare hazards. Furthermore, for each clinical entity and welfare hazard, the duration of the impact on animal welfare within each clinical entity or hazard was agreed among the experts.

#### 2.3.2. Disease Occurrence

Prevalence or incidence data from an endemic situation with the disease in question was extracted from the literature (see below) and constituted the baseline data for disease frequency. Disease occurrence was calculated as the total number of events per year per population unit for a given clinical entity/welfare hazard in a given animal group. The following groups of animals were used: cows (female animals > 2 years), heifers 1–2 years, heifer calves <1 year, bull calves <1 year, and foetuses (animals preterm) composed the cattle group; sows (females > 201 days), boars (>201 days), finishers (99–201 days), weaners (25–98 days), and piglets (0–25 days) were included for the pigs.

BVD: The calculations for postnatal infections and foetal infections were performed separately. For postnatal infections and in endemic situations with no systematic control programme in place, the annual incidence risk of infection has been estimated at 34%, which was shown to be similar across age groups [12]. This annual incidence risk was then converted to an annual incidence risk for each clinical entity (i.e., the effective incidence risk (EIR), where the occurrence of the specific clinical entity was estimated based on the occurrence of infection and information in literature). As some clinical entities (e.g., retained placenta and abortions) only occur if the animal is infected during certain risk periods, it is necessary to take this risk period into account. As the calculations addressing risk periods are easier to perform using rates, the incidence risk was changed to incidence rate, and then risk periods were addressed and the rate was re-calculated to an annual incidence risk. Therefore, the following steps were used:

- annual incidence risk of infection obtained from the literature;
- risk of clinical entity occurring if animal is infected during the risk period, based on expert opinion (H.H.);
- calculation of annual incidence risk of clinical entity (a × b);
- calculation of annual incidence rate of clinical entity (i.e., clinical events per animal year at risk): calculated from c) using the formula
  $$I_{rate}=\frac{-\ln\left(1-I_{risk}\right)}{t},$$
  where *t* is the risk period, and *I_risk_* is the annual incidence risk (p. 84 in [13]);
- estimation of the risk period based on expert opinion (H.H.), measured in years;
- calculation of the effective incidence rate (*EIR*) as
  $$EIR=I_{rate}\times t\;years,$$
  in other words, the actual rate given the animal is not at risk for an entire year is therefore expressed as “effective” IR or *EIR*;
- conversion of *EIR* to effective annual incidence risk (EAIR) using:
  $$I_{risk}=1-e^{I_{rate}\times t}$$
  (Formula 6.2 in [13]);
- total number of events = population size (n = 1,000,000) multiplied by EAIR.

For foetal infections, a more direct calculation was used. It has been estimated that the risk of foetal infection during the first 3 months of pregnancy is approximately 3%, i.e. the risk of a calf being persistently infected (PI) is 3% [12]. In order to address the risk of congenital defects being induced during a period of approximately 2 months during pregnancy, a risk of 2% was used for these animals. After addressing the risk periods, the EIR were then obtained by multiplying these incidence risks with an estimation of the probability that the clinical entity would occur if infected.

IBR: There were no estimates of the annual incidence risk of IBR infection at animal level in the Danish cattle population prior to the initiation of a control and eradication programme in 1985. Therefore, the best expert guess was based on (a) information from 1985 [14] about the number of IBR test-positive dairy cattle herds, the number of dairy cattle herds, and the population of dairy cattle and (b) an estimate of the within-herd incidence risk in test-positive herds in 1995, assuming this would be similar to the within-herd incidence risk in 1985. The estimation was as follows: a recurrent outbreak of IBR in 1995 (after having been eradicated from the Danish cattle population in 1992) in 61 dairy herds comprised 1560 test-positive cattle [15]. An estimate of the population at risk in the 61 dairy herds was based on information about the total number of cattle (2,090,373) and the total number of herds (30,250) [16]. This gives an average herd size of 2,090,373 ÷ 30,250 = 69.1 cattle. The population at risk in the 61 dairy herds was therefore estimated to be 61 × 69.1 = 4215 cattle. Thus, in 1995, the estimated annual animal-level incidence risk of being test positive was 1560 cases ÷ 4215 = 0.37. In 1985, there were approximately 31,773 dairy herds with 896,358 dairy cows, and the number of IBR test-positive dairy herds was 2667. Based on the above assumptions, the annual incidence risk of IBR infection in 1985 was estimated to be:

*I_risk_* = (2667 herds × (896,358 cows ÷ 31,773 dairy herds) × 0.37 cases per cow) ÷ (896,358 cows) = 0.031 or 3.1%. Based on the literature, it was assumed that all age groups were at the same risk of infection.

The number of clinical events was then calculated based on the probability of the presence of the three clinical entities among those infected (cf. Table 1), i.e., there was a 90% probability of the clinical entity acute phase 1, a 10% probability of the clinical entity acute phase 2, and a 5% probability of the clinical entity abortion.

MAP: MAP infections were only deemed to be relevant for adult cattle. The annual incidence risk of clinical cases was reported as 3.6% prior to the establishment of a control programme [17]. This was used to calculate the total number of events per year by multiplying it by the population size for the clinical entity Stage III MAP infection. However, not all cattle will proceed to the clinical entity Stage IV, so the effective annual incidence rate was considered to be only 50% of the effective annual incidence rate for cattle in Stage III.

Aujeszky’s disease: The following age groups were used for pigs: (a) piglets infected in utero; (b) piglets < 3 weeks of age; (c) weaners and finisher pigs (>3 weeks); and (d) adult pigs.

The annual incidence risk of Aujeszky’s disease virus infection in Danish pig herds prior to the initiation of the eradication programme in 1982 was reported to be 90 positive herds (Bitsch, 1984) out of a total of 55,000 pig holdings, yielding an annual incidence risk of 0.16%, which was similar across all age groups. Morbidity has been reported to be generally high at 100% [8], while mortality is considered highest in young animals (e.g., piglets < 3 weeks = 100%) and declining with increasing age (e.g., weaners and finishers = 50%, adult pigs < 5%). Adult pigs have a varying morbidity ranging from 10–30%. Based on these estimates, the effective incidence risk prior to eradication of Aujeszky’s disease virus from the Danish population was calculated using an annual incidence risk of 0.16% multiplied by the estimated age-group-specific prevalence of animals showing clinical manifestations among infected pigs.

PRRS: There are four clinical entities for which frequency estimates are needed for PRRS: reproductive issues caused by chronic re-exposure in sows, and acute infection in each of the age groups sows/boars, nursery piglets, and weaners/finishers. Estimating the required frequencies is greatly facilitated by [18], which provides direct estimates for the proportion of animals in Denmark affected with clinical signs (relating to endemic disease), i.e., 5% for weaners/finishers and 10% for sows (note: the table given by de Paz [18] states that this is a prevalence estimate, but given the description of the numbers in the text, the interpretation appears to be closer to an incidence risk). These estimates are therefore used directly as the effective incidence risk for chronic re-exposure in sows (0.05) and acute infection in weaners/finishers (0.1). For acute clinical disease in piglets, it was assumed that the proportion of nursery piglets exposed to disease was the same as the chronic re-infection rate in sows, and the approximate ratio of morbidity between sows (reported as 100%) and nursery piglets (reported as up to 80%) was used to adjust the effective incidence risk to 75% of 0.01 = 0.075. For acute clinical disease in adult pigs, an average morbidity estimate of 27.5% (based on the 5–50% range of [19]) was combined with the expert assumption that 1% of farms experience an epidemic outbreak annually, thus arriving at an effective incidence risk of 0.00275.

Welfare hazards. The calculation of EAIR for the ten welfare hazards was primarily based on expert judgements.

- No access to water—cattle. This was considered to occur for 1% of animals annually.
- Broken femur—cows and sows. This was considered to have an EAIR of 0.1%.
- Lying on a concrete floor with no bedding material was considered to occur with an EIR of 1% of dairy cows per day.
- Weather conditions are too hot. Heat stress generally occurs at 22 °C, and it is considered to affect the majority of dairy cattle when temperatures rise above 25 °C. Therefore, we deemed that heat stress occurs in 90% of dairy cows when the temperature reaches 25 °C.
- Separation of cow and calf was assumed to occur once for 95% of calves born to dairy cows, and each cow was assumed to have one calf per year, so the total number of events is equal to the population size × 0.95.
- Weaning piglets at 28 days occurs once in 95% of piglets born, so the total number of events is equal to the population size × 0.95.
- Tail biting has a prevalence of 3% in weaners and finishers, so the total number of events is equal to the population size × 0.03.
- Crating of sows. It was assumed that 95% of the sow population farrow 2.5 times per year, so the total number of events is 2.5 × population size × 0.95.
- Feed restriction occurs 2.5 times per year in 98% of the sow population, so the total number of events is 2.5 × population size × 0.98.

For each disease entity, the number of annual events was estimated by multiplying the incidence risk with the population size. In all cases (both disease entities and welfare hazards), the population size used was 1,000,000 for each age group.

### 2.4. Aggregation of Suffering Scores Based on Severity, Duration and Occurrence

Occurrence was aggregated with severity and duration to obtain the total “suffering” score for the specific clinical entity and animal group, i.e., the suffering score was Risk × Time × Severity. The risk is equal to the number of cases ÷ population at risk. Time is measured in units of days. Severity is measured on an ordinal scale of 0 to 10, where 0 = not severe and 10 = maximum severity. This analogue scale represents the human experts’ subjective perception of the “amount” of, e.g., pain, dullness, weakness, discomfort, and dizziness associated with the condition.

The suffering scores were estimated using Monte Carlo approximation with 1000 iterations per disease. For each disease and clinical entity, suffering scores were aggregated based on the severity scores (from each expert), duration, and occurrence. Aggregation was performed as follows: for a given disease and clinical entity, a random theoretical animal of a given species and age group was selected. Whether or not the animal had the clinical entity was randomly chosen based on the disease occurrence (total number of events per year). How long the animal would suffer from the disease was then randomly chosen based on the distribution given by the experts. This distribution was derived from the mode and 2.5/97.5 percentiles elicited by the experts and mapped into a triangular distribution fitted using the triangle package [20] for R version 3.6.3 (R Foundation for Statistical Computing, Austria, Vienna).

The total suffering was then calculated by Monte Carlo integration, resulting in an estimated distribution of suffering per clinical entity and expert. The combined expert score was used if no systematic effect of expert was found.

## 3. Results

Animal Welfare Scoring and Assessment

**Figure 1:**
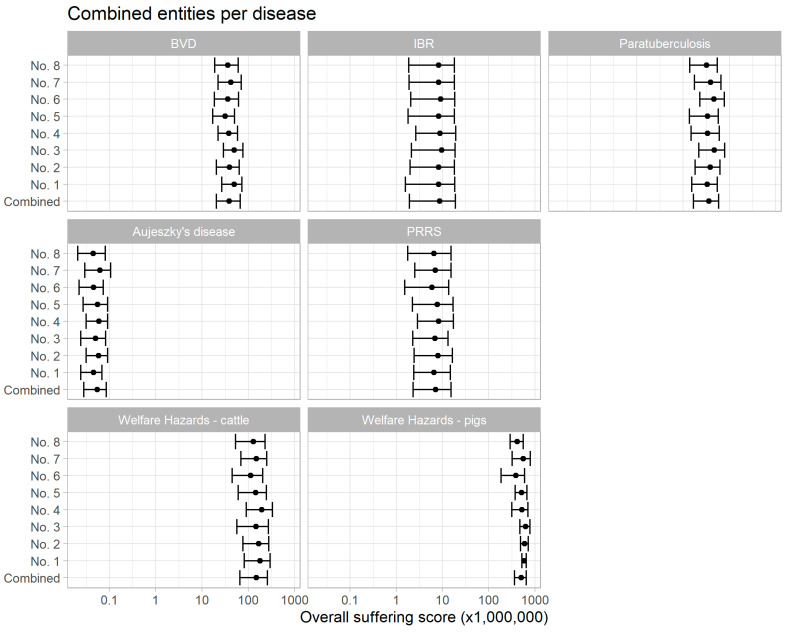
The aggregated suffering scores (2.5, 50, and 97.5 percentiles) given by eight experts (specified as No. 1–No. 8).

**Figure 2:**
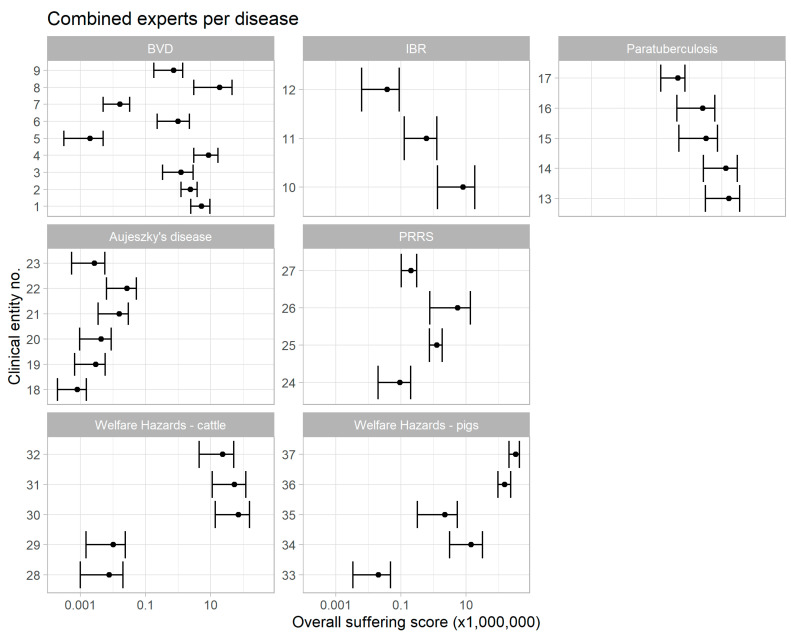
Aggregated suffering scores (2.5, 50, and 97.5 percentiles) by disease condition. The disease entities and welfare hazards are numbered as follows: 1–9, BVD: (1) transient infection; (2) transient infection with erosions; (3) comorbidity (mastitis, retained placenta); (4) comorbidity (respiratory disease); (5) repeat breeding; (6) abortion; (7) congenital defects; (8) persistent infection (unthrifty animals); (9) persistent infection: mucosal disease; 10–12, IBR: (10) acute phase, respiratory version; (11) acute phase, gastro-intestinal version; (12) abortion; 13–17, MAP infection: (13) stage III with chronic wasting; (14) stage III with intermittent diarrhoea; (15) stage III with emaciation; (16) stage IV with pipe stream diarrhoea; (17) stage IV with lethargy; 18–23, Aujeszky’s disease: (18) encephalitis (weak piglets); (19) encephalitis (lethargic piglets); (20) encephalitis—weaners and finishers; (21) encephalitis—adults; (22) respiratory signs—finishers; (23) reproductive problems; 24–27, PRRS: (24) re-exposure in sows; (25) acute infection in piglets; (26) acute infection in weaners and finishers; (27) acute infection in sows and boars; 28–32, welfare hazards in cattle: (28) no access to water; (29) broken femur; (30) lying on concrete floor with no bedding material; (31) weather conditions are too hot; (32) separation of cow and calf; 33–37, welfare hazards in pigs: (33) broken femur; (34) weaning of piglets; (35) tail biting; (36) crating of sows; (37) feed restriction for sows.

Table 3 shows the distributions of the duration of each clinical entity for each disease and welfare hazard as agreed by the experts through the EKE. These were combined with the calculated number of events for each clinical entity (Table 4) and the combined severity scores from the EKE (Table 5). The distribution of scores for the eight experts are shown in Figure 1. Due to the general overlap, and because no expert seemed to be systematically different from others, the combined scores were used for all subsequent reporting. The severity scores for all clinical entities and welfare hazards are shown in Figure 2. The summarised scores are also shown in Table 6, and the ranked means of the summarised severity scores are as follows (from lowest to highest): broken femur in cattle < broken femur in pigs < no access to water (cattle) < Aujeszky’s disease < tail biting < PRRS < IBR < weaning of piglets < separation of cow and calf < weather conditions are too hot (for cattle) < MAP infection and BVD < cattle lying on concrete floor with no bedding material < crating of sows < feed restriction. These conditions are for the average affected individual for the average duration that this animal would be expected to endure the condition over a year, summarised for the population.

## 4. Discussion

To the best of our knowledge, this study is the first to attempt to quantify the impact of specific diseases on animal welfare. For example, it appears that Aujeszky’s disease has less impact on pigs than PRRS at population level, while our ranking shows that, for cattle, BVD and MAP are worse than IBR in the endemic situation with no organised control effort/programme. If these assessments are considered valid, the data can be aggregated to country level and the effect of disease control on animal welfare can be estimated and assessed.

In general, we found some surprising results, as clinical entities with painful clinical signs and a short duration had a relatively small effect on animal welfare, although a potentially high prevalence must also be taken into consideration. Therefore, disease entities with a short duration and low prevalence result in a smaller impact on animal welfare, whereas clinical entities with a long duration will have a greater impact at population level. Another finding is the high impact of some of the non-infectious welfare hazards due to their high prevalence (e.g., weaning of piglets and separation of calf and cow). In the present paper, calculations were based on a denominator of 1,000,000 animals to allow for comparison between populations. However, estimations can also be made at country level and to allow for comparison across populations of pigs and cattle. For example, the Danish pig population of >20 million pigs produced annually is much bigger than the cattle population of around 1 million.

The numbers may be used to compare diseases, welfare hazards, and for comparison between countries. However, caution should be advised. Firstly, there are limited data on the severity of clinical signs and their distributions available in the literature, and the distributions are often only vaguely described, as are the duration and frequency. Therefore, the aggregation of suffering scores with duration and frequency results in major uncertainty. We therefore used EKE as a tool. EKE is influenced by the views and experiences of the experts involved, which, in turn, are affected by their field experience. Therefore, we used the Delphi method, firstly with individual assessments followed by group discussions, still allowing for individual variation. Some variation was indeed found among experts, but no expert seemed to be systematically more uncertain, score diseases higher, or stand out in any other way (Figure 1). Therefore, we chose to combine the estimates from different experts and allow the model to absorb the uncertainty at expert level. Other experts may provide different results, and the model is subject to uncertainty at this level. For example, the literature reviews may not have included all relevant clinical signs, especially the less severe ones. For example, we included “repeat breeding” for BVD, although it is not a severe condition. This was included because breeding can induce stress for cattle, but breeding is also a common event and other causes of repeat breeding exist. Therefore, it could be argued whether or not this should have been included. The same applies to other conditions. For MAP infections, we did not include, e.g., “bottle jaw”, where oedema may be present around the jaws. However, this was included in the already described entities and may not have been any more serious than the already described and more predominant features “chronic wasting” and “intermittent diarrhoea”, and it was therefore deemed to be unnecessary to include this separately. In general, we do not specifically include the animals’ physiological or hormonal responses directly, because no such information was found in the extensive literature searches that we performed. However, in principle, such information is included through the EKE.

Despite these limitations, the model presented here provides an insight into how animal welfare consequences of infectious diseases can be aggregated and quantified at population level. In terms of pig diseases, Aujeszky’s disease clearly has less impact on animal welfare than PRRS, which is likely to be due to the prolonged nature of PRRS, despite the disease entity “encephalitis” associated with Aujeszky’s disease being given relatively high mode scores of 7 out of 10 for severity in piglets and scores of 6.5–7 in other pigs. However, the most likely duration of 1 day in piglets resulted in an aggregated score that was relatively low, even though the severity for PRRS was not much lower (5.5 and 4 for nursery pigs and weaners and finishers, respectively), while a longer duration of 7 days (nursery pigs) and 6 days (weaners and finishers) had a major impact. The main drivers of the aggregated scores are therefore duration and population size. In cattle, these combinations resulted in the following ranking of diseases: BVD and MAP > IBR, which is logical, because BVD in the endemic situation is highly prevalent (for transiently infected animals) and of long duration (for persistently infected animals), whereas MAP is of relatively low prevalence but very long duration. The same features (high prevalence and long duration) also have an impact on the non-infectious welfare hazards; a lack of access to water and broken femurs are rare and of short duration and therefore do not have a major impact at population level. However, weather conditions that are too hot can have a long duration and affect the majority of the cattle population, while feed restriction, crating of sows, and early weaning of piglets affect a large proportion of the pig population and for a very long time. Even though the severity may not rank highly, the long duration is a major driver and gives these hazards a similar or greater impact than PRRS. We choose some infections which have been relevant to Danish conditions for the past 40 years. However, the method can also be applied to other infectious diseases. It may be applied to quantification of animal welfare burdens in initiatives such as Global Burden of Animal Diseases [21]. In many of the studies of eradication programmes, animal welfare is mentioned as one of several aspects to be taken into account (e.g., [22,23,24]); using the methods of the current study, this aspect may be quantified and used in making decisions on eradication strategies.

## 5. Conclusions

We developed an analysis method that can be used to assess the impact of infectious diseases on animal welfare in cattle and pigs at population level. The model suggests that the impact ranking for cattle is: BVD and MAP > IBR, and for pigs: PRRS > Aujeszky’s disease. The impact of BVD may on average be similar to 1 week of weather conditions that are too hot per year, and the impact of PRRS is similar to feed restriction, crating of sows, and weaning of piglets. However, major sources of uncertainty exist, and these should be taken into account when the results are interpreted.

## Figures and Tables

**Table 1:** Typical clinical signs stratified by clinical disease entities and age group for each of the five diseases ^1^, as described in the literature.

Disease	Clinical Entity	Age Group	Typical Signs
BVD	Transient infection	Calves	Diarrhoea
		Heifers	Diarrhoea
		Cows	Diarrhoea
	Transient infection with erosions	Calves	Mucosal erosions
		Heifers	Mucosal erosions
		Cows	Mucosal erosions
	Co-morbidity	Heifers	Retained placenta
		Cows	Mastitis
		Cows	Retained placenta
		Calves	Respiratory disease, diarrhoea
		Heifers	Respiratory disease, diarrhoea
	Repeat breeding	Heifers	Subclinical
		Cows	Subclinical
	Abortion	Heifers	Abortion early or late after infection
		Cows	Abortion early or late after infection
	Congenital defects	Newborn	Miscellaneous malformations incl. congenital tremor and weak calves
	PI: unthriftiness	Calves & heifers	Weight loss
	PI: mucosal disease	Calves & heifers	Severe diarrhoea and erosion
IBR	Acute phase 1	Calves, heifers & cows	Reduced appetite, dullness, salivation, nasal and ocular discharge, lachrymation, conjunctivitis, rapid respiration, coughing and pyrexia. May lead to death
	Acute phase 2		Diarrhoea and dehydration
	Abortion	Cows	Abortion during 2nd and 3rd trimesters
MAP	Stage III	Cows	Weight loss/poor condition (BCS 1–2)/chronic wasting
			Intermittent diarrhoea
			Emaciation (BCS 0–1)
	Stage IV	Cows	Pipe stream diarrhoea
			Lethargy
Aujeszky’s	Encephalitis	Piglets infected in utero	Weak piglets; shaking/shivering/sudden death
	Encephalitis	Piglets (<3 weeks)	Lethargy; weakness/appetite loss; incoordination/convulsions (vomiting, diarrhoea) incl. febrile response (up to 42℃)
	Encephalitis	Weaners & finisher pigs (>3 weeks)	Loss of appetite; somnolence; trembling/convulsions; paralysis; high temperature (up to 42℃)
	Encephalitis	Adult pigs	Incoordination of hind limbs and febrile response (up to 42 °C)
	Respiratory signs	Weaners & finishers (>3 weeks)	Sneezing/nasal discharge; coughing; dyspnoea
	Reproduction		Vaginal discharge; mummification; agalactia
PRRS	Re-exposure	Sows	Reproductive problems incl. abortion, still-birth and return to service
	Acute infection	Sows & boars	Anorexia, fever, lethargy, respiratory difficulties, cyanosis
		Nursery piglets	Poor growth, anorexia, fever, respiratory distress, diarrhoea, anaemia, congenital abnormalities, weakness, ataxia, haemorrhage, immunomodulation
		Weaners & finishers	Transient anorexia, respiratory disorders and discolouration of the ears

^1^ BVD: bovine virus diarrhoea; IBR: infectious bovine rhinotracheitis; MAP: *Mycobacterium avium* subsp. *paratuberculosis*; PRRS: porcine reproductive and respiratory syndrome. PI: Persistently infected. BCS: Body condition score.

**Table 2:** Scenario description of ten welfare hazards occurring in dairy cattle and pig production used for comparison.

Hazard	Age Group	Scenario
No access to water—cattle	Cows	Dairy cattle left with no access to water due to broken pipes
Broken femur—cattle	Cows	Dairy cow falls on slippery floor and is left until euthanised
Broken femur—pigs	Sows	Sow falls on slippery floor with other sows and is pushed around
Lying on concrete floor with no bedding material	Cows	Dairy cows resting in a free stall environment
Weather conditions are too hot	Cows	Warm summer days in Denmark (>25 °C) interrupted by cooler nights with no access to shade during the daytime
Separation of cow and calf	Calves	Calf separated within 24 h of birth
Weaning of piglets	Piglets	Piglets weaned 3–4 weeks after birth
Tail biting	Weaners and finishers	Ongoing and severe tail biting (part of tail bitten off, blood present)
Crating of sows	Sows	Sows crated in farrowing section (from 1 week prior to farrowing)
Feed restriction	Sows	Feed provision reduced to 30% of ad lib during gestation. Sows are housed together, but most often feed provision is individual

**Table 3:** Distribution (mode and 2.5/97.5 percentiles (p)) of the duration (days) of clinical entities based on literature and expert knowledge elicitation for the five diseases ^1^ and ten non-infectious welfare hazards.

Disease	Clinical Entity	Age Group	Typical Signs	2.5p	Mode	97.5p
BVD	Transient infection	Calves	Diarrhoea	5	10	35
		Heifers & cows	Diarrhoea	5	10	35
	Transient infection with erosions	Calves	Mucosal erosions	8	14	45
		Heifers & cows	Mucosal erosions	8	14	45
	Co-morbidity	Calves	Respiratory disease, diarrhoea	2	3	14
		Heifers	Respiratory disease, diarrhoea	7	20	56
		Heifers & cows	Mastitis, retained placenta	7	20	56
	Repeat breeding	Heifers	Subclinical	1	4	6
		Cows	Subclinical	1	4	6
	Abortion	Heifers & cows	Abortion early or late after infection	1	4	35
	Congenital defects	Newborn	Miscellaneous malformations incl. congenital tremor and weak calves	1	2	3
	PI: animals unthrifty	Calves & heifers	Weight loss	100	400	700
	PI: mucosal disease	Calves & heifers	Severe diarrhoea and erosion	4	21	45
IBR	Acute phase	Calves, heifers & cows	Reduced appetite, dullness, salivation, nasal and ocular discharge, lachrymation, conjunctivitis, rapid respiration, coughing and pyrexia. May lead to death	7	42	100
		Calves, heifers & cows	Diarrhoea and dehydration	7	49	100
	Abortion	Cows	Abortion during 2nd and 3rd trimesters	0	14	30
MAP	Stage III	Cows	Weight loss/poor condition (BCS 1–2)/chronic wasting	15	120	240
		Cows	Intermittent diarrhoea	15	120	240
		Cows	Emaciation (BCS 0–1)	1	10	60
	Stage IV	Cows	Pipe stream diarrhoea	1	10	60
		Cows	Lethargy	1	4	5
Aujeszky’s	Encephalitis	Piglets infected in utero	Weak piglets, shaking/shivering/sudden death	0.1	1	2
	Encephalitis	Piglets (<3 weeks)	Lethargy, weakness/appetite loss, incoordination/convulsions (vomiting, diarrhoea) incl. febrile response (up to 42 °C)	1	4	8
	Encephalitis	Weaners & finishers (>3 weeks)	Loss of appetite, somnolence, trembling/convulsions, paralysis, high temperature (up to 42 ℃)	1	4	8
	Encephalitis	Adult pigs	Incoordination of hind limbs and febrile response (up to 42 °C)	1.5	6	14
	Respiratory signs	Weaners & finishers (>3 weeks)	Sneezing/nasal discharge, coughing, dyspnoea	1	4	8
	Reproduction		Vaginal discharge, mummification, agalactia	1	4	8
PRRS	Re-exposure	Sows	Reproductive problems incl. abortion, still-birth, and return to service	2	5	14
	Acute infection	Sows & boars	Anorexia, fever, lethargy, respiratory difficulties, cyanosis	1	1	1
		Nursery piglets	Poor growth, anorexia, fever, respiratory distress, diarrhoea, anaemia, congenital abnormalities, weakness, ataxia, haemorrhage, immunomodulation	2	7	28
		Weaners & finishers	Transient anorexia, respiratory disorders and discolouration of the ears	5	6	7
Welfare Hazards—cattle	No access to water—cattle	Cows	Scenario: dairy cattle left with no access to water due to broken pipes	0.25	1	3
	Broken femur—cattle	Cows	Scenario: dairy cow falls on slippery floor and is left until euthanised	0.1	0.5	3
	Lying on concrete floor with no bedding material	Cows	Scenario: dairy cows resting in a free stall environment	90	1000	3000
	Weather conditions are too hot	Cows	Scenario: warm summer days (>25 ℃) interrupted by cooler nights. No access to shade during the daytime	1	7	21
	Separation of cow and calf	Calves	Scenario: calf separated within 24 h of birth	1	5	12
Welfare hazards—pigs	Broken femur—pigs	Sows	Scenario: sow falls on slippery floor with other sows and is pushed around	0.25	1	6
	Weaning of piglets	Piglets	Scenario: piglets are weaned 3–4 weeks after birth	1	3	8
	Tail biting	Weaners & finishers	Scenario: ongoing severe tail biting (part of tail bitten off, blood present)	1	5	28
	Crating of sows	Sows	Scenario: sows are crated in farrowing section (from 1 week prior to farrowing)	28	32	45
	Feed restriction	Sows	Scenario: feed provision is reduced to 30% of ad lib during gestation. Sows are housed together, but most often feed provision is individual	80	85	95

^1^ Aujeszky’s: Aujeszky’s disease; BVD: bovine virus diarrhoea; IBR: infectious bovine rhinotracheitis; MAP: *Mycobacterium avium* subsp. *paratuberculosis*; PRRS: porcine reproductive and respiratory syndrome. PI: Persistently infected. BCS: Body condition score.

**Table 4:** Annual incidence risk (IR) and total number of events (TNE) per 1 million animals for each clinical entity for the five diseases ^1^ and ten non-infectious welfare hazards.

Disease	Clinical Entity	Age Group	Typical Signs	IR	TNE
BVD	Transient infection	Calves	Diarrhoea	0.03125	31,250
		Heifers	Diarrhoea	0.034	34,000
		Cows	Diarrhoea	0.034	34,000
	Transient infection with erosions	Calves	Mucosal erosions	0.006243	6243
		Heifers	Mucosal erosions	0.0068	6800
		Cows	Mucosal erosions	0.0068	6800
	Co-morbidity	Heifers	Retained placenta	0.001710	1710
		Cows	Mastitis	0.0408	40,800
		Cows	Retained placenta	0.001710	1710
		Calves	Respiratory disease, diarrhoea	0.04691	46,908
		Heifers	Respiratory disease, diarrhoea	0.017	17,000
	Repeat breeding	Heifers	Subclinical	0.001421	1421
		Cows	Subclinical	0.001421	1421
	Abortion	Heifers	Abortion early or late after infection	0.013507	13,507
		Cows	Abortion early or late after infection	0.013507	13,507
	Congenital defects	Newborn	Miscellaneous malformations incl. congenital tremor and weak calves	0.002	2000
	PI: animals unthrifty	Calves & heifers	Weight loss	0.012	12,000
	PI: mucosal disease	Calves & heifers	Severe diarrhoea and erosion	0.0036	3600
IBR	Acute phase	Calves, heifers & cows	Reduced appetite, dullness, salivation, nasal and ocular discharge, lachrymation, conjunctivitis, rapid respiration, coughing and pyrexia, may lead to death	0.0279	27,900
		Calves, heifers & cows	Diarrhoea and dehydration	0.0031	3100
	Abortion	Cows	Abortion during 2nd and 3rd trimesters	0.0007647	765
MAP	Stage III	Cows	Weight loss/poor condition (BCS 1–2)/chronic wasting	0.036	36,000
		Cows	Intermittent diarrhoea	0.036	36,000
		Cows	Emaciation (BCS 0–1)	0.018	18,000
	Stage IV	Cows	Pipe stream diarrhoea	0.018	18,000
		Cows	Lethargy	0.018	18,000
Aujeszky’s	Encephalitis	Piglets infected in utero	Weak piglets, shaking/shivering/sudden death	0.0001097	110
	Encephalitis	Piglets (<3 weeks)	Lethargy, weakness/appetite loss, incoordination/convulsions (vomiting, diarrhoea) incl. febrile response (up to 42 °C)	0.000146	146
	Encephalitis	Weaners & finishers (>3 weeks)	Loss of appetite, somnolence, trembling/convulsions, paralysis, high temperature (up to 42 °C)	0.00056	560
	Encephalitis	Adult pigs	Incoordination of hind limbs and febrile response (up to 42 °C)	0.0008113	811
	Respiratory signs	Weaners & finishers (>3 weeks)	Sneezing/nasal discharge, coughing, dyspnoea	0.0001097	110
	Reproduction		Vaginal discharge; mummification; agalactia	0.00032	320
PRRS	Re-exposure	Sows	Reproductive problems incl. abortion, still-birth, and return to service	0.00275	2750
	Acute infection	Sows & boars	Anorexia, fever, lethargy, respiratory difficulties, cyanosis	0.1	100,000
		Nursery piglets	Poor growth, anorexia, fever, respiratory distress, diarrhoea, anaemia, congenital abnormalities, weakness, ataxia, haemorrhage, immunomodulation	0.075	75,000
		Weaners & finishers	Transient anorexia, respiratory disorders and discolouration of the ears	0.05	50,000
Welfare hazards— cattle	No access to water—cattle	Cows	Scenario: dairy cattle left with no access to water due to broken pipes		5600
	Broken femur—cattle	Cows	Scenario: dairy cow falls on slippery floor and is left until euthanised	0.001	1000
	Lying on concrete floor with no bedding material	Cows	Scenario: dairy cows resting in a free stall environment	0.01	10,000
	Weather conditions are too hot	Cows	Scenario: warm summer days (>25 °C) interrupted by cooler nights. No access to shade during the daytime.	0.9	900,000
	Separation of cow and calf	Calves	Scenario: calf separated within 24h of birth	0.95	950,000
Welfare hazards—pigs	Broken femur—pigs	Sows	Scenario: sow falls on slippery floor with other sows and is pushed around	0.001	1000
	Weaning of piglets	Piglets	Scenario: piglets are weaned 3–4 weeks after birth	0.95	950,000
	Tail biting	Weaner & finisher pigs	Scenario: ongoing severe tail biting (part of tail bitten off, blood present)	0.03	30,000
	Crating of sows	Sows	Scenario: sows are crated in farrowing section (from 1 week prior to farrowing)	0.95	950,000
	Feed restriction	Sows	Scenario: feed provision is reduced to 30% of ad lib during gestation. Sows are housed together, but most often feed provision is individual	0.98	980,000

^1^ Aujeszky’s: Aujeszky’s disease; BVD: bovine virus diarrhoea; IBR: infectious bovine rhinotracheitis; MAP: *Mycobacterium avium* subsp. *paratuberculosis*; PRRS: porcine reproductive and respiratory syndrome. PI: Persistently infected. BCS: Body condition score.

**Table 5:** Combined distributions of severity for clinical entities of five diseases ^1^ and ten non-infectious welfare hazards assessed through expert knowledge elicitation. The tabulated severity scores present the median of the scores from the eight experts for each of the percentiles 2.5, median, and 97.5.

Disease	Clinical Entity	AGE Group	Typical Signs	2.5p	Median	97.5p
BVD	Transient infection	Calves	Diarrhoea	1	3	5
		Heifers & cows				
	Transient infection with erosions	Calves	Mucosal erosions	3.5	5	7
		Heifers & cows				
	Co-morbidity	Calves & heifers	Respiratory disease, diarrhoea	3	4.5	7
		Cows	Mastitis, retained placenta	2	5	6
	Repeat breeding	Heifers & cows	Subclinical	0	0	0.5
	Abortion	Heifers & cows	Abortion early or late after infection	1	2.5	4
	Congenital defects	Newborn	Miscellaneous malformations incl. congenital tremor and weak calves	1.5	4	7
	PI: animals unthrifty	Calves & heifers	Weight loss	1	4	7
	PI: mucosal disease	Calves & heifers	Severe diarrhoea and erosion	7	8	9.5
IBR	Acute phase	Calves, heifers & cows	Reduced appetite, dullness, salivation, nasal and ocular discharge, lachrymation, conjunctivitis, rapid respiration, coughing and pyrexia. May lead to death	2.5	6	8
		Calves, heifers & cows	Diarrhoea and dehydration	2	3.5	5
	Abortion	Cows	Abortion during 2nd and 3rd trimesters	1	3	5
MAP	Stage III	Cows	Weight loss/poor condition (BCS 1–2)/chronic wasting	2	3.5	5
		Cows	Intermittent diarrhoea	1	3	4.5
		Cows	Emaciation (BCS 0–1)	5	7.5	8.5
	Stage IV	Cows	Pipe stream diarrhoea	3.5	5.5	7
		Cows	Lethargy	6	8	9
Aujeszky’s	Encephalitis	Piglets infected in utero	Weak piglets, shaking/shivering/sudden death	5	7	8.5
	Encephalitis	Piglets (<3 weeks)	Lethargy, weakness/appetite loss, incoordination/convulsions (vomiting, diarrhoea) incl. febrile response (up to 42 °C)	4.5	6	8
	Encephalitis	Weaners & finishers (>3 weeks)	Loss of appetite, somnolence, trembling/convulsions, paralysis, high temperature (up to 42 °C)	5	7	9
	Encephalitis	Adult pigs	Incoordination of hind limbs and febrile response (up to 42 °C)	5	6.5	8
	Respiratory signs	Weaners & finishers (>3 weeks)	Sneezing/nasal discharge, coughing, dyspnoea	3	5	6
	Reproduction		Vaginal discharge, mummification, agalactia	1	2	3
PRRS	Re-exposure	Sows	Reproductive problems incl. abortion, still-birth, and return to service	2	4.5	7
	Acute infection	Sows & boars	Anorexia, fever, lethargy, respiratory difficulties, cyanosis	1	2	3
		Nursery piglets	Poor growth, anorexia, fever, respiratory distress, diarrhoea, anaemia, congenital abnormalities, weakness, ataxia, haemorrhage and immunomodulation	2.5	5.5	8.5
		Weaners & finishers	Transient anorexia, respiratory disorders and discolouration of the ears	2.5	4	6
Welfare hazards— cattle	No access to water—cattle	Cows	Scenario: dairy cattle left with no access to water due to broken pipes	1	5	9
	Broken femur—cattle	Cows	Scenario: dairy cow falls on slippery floor and is left until euthanised	6	7.5	9.5
	Lying on concrete floor with no bedding material	Cows	Scenario: dairy cows resting in a free stall environment	3	5	6.5
	Weather conditions are too hot	Cows	Scenario: warm summer days (>25 °C) interrupted by cooler nights. No access to shade during the daytime.	3.5	5.5	8
	Separation of cow and calf	Calves	Scenario: calf separated within 24h of birth	2	4	6
Welfare hazards—pigs	Broken femur—pigs	Sows	Scenario: sow falls on slippery floor with other sows and is pushed around	6	8	9.5
	Weaning of piglets	Piglets	Scenario: piglets are weaned 3–4 weeks after birth	2	4	5
	Tail biting	Weaners & finishers	Scenario: ongoing severe tail biting (part of tail bitten off, blood present)	4	6	8
	Crating of sows	Sows	Scenario: sows are crated in farrowing section (from 1 week prior to farrowing)	3	4	6.5
	Feed restriction	Sows	Scenario: feed provision is reduced to 30% of ad lib during gestation. Sows are housed together, but most often feed provision is individual	2.5	4.5	5

^1^ Aujeszky’s: Aujeszky’s disease; BVD: bovine virus diarrhoea; IBR: infectious bovine rhinotracheitis; MAP: *Mycobacterium avium* subsp. *paratuberculosis*; PRRS: porcine reproductive and respiratory syndrome. PI: Persistently infected. BCS: Body condition score.

**Table 6:** Suffering scores for four infectious diseases in cattle and two infectious diseases in pigs, five welfare hazards in cattle, and five welfare hazards in pigs. The suffering scores pertain to a population of 1 million animals in a year, but because the durations vary, the effect of short-term incidents (such as no access to water) can be less than for long-term incidents (such as lying on a concrete floor).

Disease/Welfare Hazard	Suffering Score (×1,000,000)		
	2.5 lower CL	Mode	97.5 upper CL
BVD	20	39	65
IBR	2.1	8.7	18
MAP	17	36	60
Aujeszky’s disease	0.028	0.055	0.086
PRRS	2.4	7.1	15
WH Cattle: No access to water	0.001	0.007	0.019
WH Cattle: Broken femur	0.002	0.01	0.024
WH Cattle: Lying on concrete floor with no bedding material	15	70	162
WH Cattle: Weather conditions are too hot	9.9	54	119
WH Cattle: Separation of cow and calf	4.0	24	54
WH Pigs: Broken femur—pigs	0.003	0.022	0.051
WH Pigs: Weaning of piglets	3.3	15	32
WH Pigs: Tail biting	0.39	2.4	5.6
WH Pigs: Crating of sows	94	159	246
WH Pigs: Feed restriction	217	344	463

Aujeszky’s: Aujeszky’s disease; BVD: bovine virus diarrhoea; IBR: infectious bovine rhinotracheitis; MAP: *Mycobacterium avium* subsp. *paratuberculosis*; PRRS: porcine reproductive and respiratory syndrome; WH: Welfare hazard; CL: confidence limit.

## Data Availability

Data are available under Supplementary Materials.

## Supplementary Materials

The following are available online at https://www.mdpi.com/article/10.3390/ani11113017/s1, Table S1: Data on expert knowledge elicitations on severity, duration and frequency, along with population sizes. Document S1: Disease fact sheets including information from extensive literature searches.
