# Supplementary material for: Application of Methods to Assess Animal Welfare and Suffering Caused by Infectious Diseases in Cattle and Swine Populations
Source: Animals (Basel). 2021 Oct 20;11(11):3017. doi: 10.3390/ani11113017 (PMC8614463; doi:10.3390/ani11113017)
Supplement: Supplementary file 1 [file animals-11-03017-s001.zip › DocumentS1_DiseaseFactSheets.pdf]

Literature reviews of clinical manifestations with qualitative assessment of impact on animal welfare for Infectious Bovine Rhinotracheitis in Cattle, Paratuberculosis in Cattle, Aujeszky's Disease in Swine and Porcine Reproductive and Respiratory Syndrome in Pigs.

Hans Houe, Jens Frederik Agger, Søren Saxmose Nielsen, Liza Rosenbaum Nielsen, Björn Forkman, Nina Dam Otten, Anne Kirstine Manly Andersen, Matt Denwood

Department of Veterinary and Animal Sciences

Section for Animal Welfare and Disease Control

Faculty of Health and Medical Sciences

University of Copenhagen

Denmark.

Preface:

With reference to the final report for the project “Infectious Livestock Diseases and Welfare”, this appendix contains summary in Danish and English and detailed English literature review for each of the diseases Infectious Bovine Rhinotracheitis in cattle, Paratuberculosis in cattle, Aujeszky's Disease in pigs and Porcine Respiratory and Reproductive Syndrome.

The Danish Centre for Animal Welfare, Danish Veterinary and Food Administration, Ministry of Environment and Food of Denmark, is acknowledged for economic support.

Content/Indhold

Literature reviews of clinical manifestations with qualitative assessment of impact on animal welfare for Infectious Bovine Rhinotracheitis in Cattle, Paratuberculosis in Cattle, Aujeszky's Disease in Swine and Porcine Reproductive and Respiratory Syndrome in Pigs. 1

Preface:.......... 2

A. Infectious Bovine Rhinotracheitis (IBR) in cattle - review of clinical manifestations. 5

A1. Summary 5

A2. Introduction of the disease 5

A3. Methods 6

A3.1. Clinical signs, severity, duration, mortality/case-fatality and impact on animal welfare of the individual 6

A3.2. Within- and between-herd prevalence and incidence in Denmark and selected countries 7

A4. Results 7

A4.1. Clinical signs, severity, duration, mortality/case-fatality and impact on animal welfare of the individual 7

A4.2. Within- and between-herd prevalence in Denmark and eight other EU member states, and with specific comparison to The Netherlands, Belgium and Ireland. 11

A5. References 18

B. Paratuberculosis in cattle - systematic search and review of clinical manifestations. 23

B1. Summary 23

B2. Introduction of the disease 23

B3. Methods 24

B3.1. Clinical signs, severity, duration, and mortality/case-fatality 24

B3.2. Within- and between-herd prevalences /incidences in Denmark, Sweden, the Netherlands and Austria 24

B4. Results 25

B4.1. Clinical signs, severity, duration, and mortality/case-fatality 25

B4.2. Within- and between-herd prevalences in Denmark, Austria, the Netherlands and Sweden.......... 28

B5. References 30

C. Aujeszky´s disease in swine - review of clinical manifestations. 35

C1. Summary.......... 35

C2. Introduction of the disease 35

C3. Methods 35

C3.1. Clinical signs, severity, duration, and mortality/case-fatality 35

C3.2. Within- and between-herd prevalences /incidences in Denmark, Germany, Sweden, The Netherlands UK and Spain 36

C4. Results 36
C4.1. Clinical signs, severity, duration, and mortality/case-fatality 36
C4.2. Within- and between-herd prevalences /incidences in Denmark 39
C4.3. Within- and between-herd prevalences in selected other countries 39
C5. References 40
D. Porcine Reproductive and Respiratory Syndrome in Pigs – systematic search and review of clinical manifestations 43
D1. Summary 43
D2. Introduction 43
D3. Methods 44
D3.1. Clinical signs, severity, duration, and mortality/case-fatality 44
D3.2. Within- and between-herd prevalence/incidence in Denmark, Sweden, the United Kingdom, the Netherlands and Spain 44
D4. Results 45
D4.1. Clinical signs, severity, duration, and mortality/case-fatality 45
D4.2. Within- and between-herd prevalence/incidence in Denmark, Sweden, the United Kingdom, the Netherlands and Spain 47
D5. References 49

A. Infectious Bovine Rhinotracheitis (IBR) in cattle - review of clinical manifestations.

By Jens Frederik Agger

A1. Summary

Infectious Bovine Rhinotracheitis (IBR) in cattle - review of clinical manifestations.

Infection with bovine herpesvirus 1 (BoHV-1) causes a variety of clinical conditions in cattle that are compromising the normal functioning, behavior and welfare of animals. The most common conditions are infectious bovine rhinotracheitis (IBR), infectious pustular vulvovaginitis (IPV) and infectious pustular balanoposthitis (IBP). This review focuses on IBR, which is the most common condition in BoHV-1 infection. The review covers the clinical signs, severity, duration and mortality, as well as the occurrence of the disease at the herd and national levels before and after commencement of eradication as well as legal aspects of importance for the control. Finally, a qualitative assessment is given of the importance of the extinction on the welfare of the cattle population in Denmark and a comparison with Ireland, Belgium and the Netherlands, which are at different stages of control. Ireland has been chosen because a control program has not yet been established, but initial steps have been taken; the frequency among dairy and beef cattle herds was 75-80% in 2015. Belgium has been chosen because a voluntary control program from 2007 was made compulsory in 2012 throughout the country (EU approved in 2014); the country is in the later stages of the control and the frequency of IBR infected herds has been reduced significantly from 52% in 2011 to 18% in 2015 but not yet eradicated. The Netherlands has been chosen because mandatory action was started in 2018 in dairy cattle and voluntarily in beef cattle; the country is in the early stages of the control and the prevalence was high with 27 % infected herds in the first quarter of 2019.

IBR typically has an acute clinical course expressed by the following 12 different clinical signs: Decreased appetite and intestinal function and milk yield, fever, increased salivation and nasal and ocular discharge, increased and superficial respiration - possibly dyspnea, short vigorous cough, dehydration, lethargy, abortion and transient diarrhea. The infection then goes into a lifelong subclinical course with a latent infection of the nerve ganglia, which makes the bovine periodically contagious. None of the clinical signs are pathognomonic to the disease, and the predictive value of the individual clinical sign is typically very low, regardless of any evidence of a high prevalence. It is the overall clinical picture, supplemented by laboratory studies for the presence of antibody or virus in a milk or blood sample, which is essential for the detection of the infection. In countries with infection, the within-herd prevalence of infection range from 10-78% and the between-herd prevalence of infected herds range from 9-100%. Lethality is generally low <10%.

A2. Introduction of the disease

Infectious bovine rhinotracheitis (IBR) is caused by Bovine herpes virus 1 (BoHV-1). The infection is lifelong (Straub 2001) and may be in an active subclinical or clinical stage with formation of antibodies or in a latent stage where the virus hides in sensory nerve ganglia mostly in the trigeminal

ganglion or pharyngeal tonsils after naso/oral infection or in the sacral ganglia after genital infection (Ackermann and Wyler 1984; Winkler et al. 2000). The latent virus may be reactivated by stressors e.g. parturition, mating, transport or treatment with corticosteroids (Muylkens et al. 2007, Raaperi et al. 2014, Straub 2001).

Bovine herpes virus 1 (BoHV-1) is believed to have been the cause of previously reported clinical disease in cattle in the 19th century in Germany denoted Bläschenausschlag or coital vesicular exanthema (CVE) and later as pustular vulvo-vaginitis in cows and heifers (IPV) and as infectious pustular balanoposthitis in bulls (IBP). According to the literature only these genital and venereal forms were known until the 1950'ies, where Schroeder and Moys (1954) reported the initial cases of IBR in large Californian dairy herds in 1953. BoHV-1 may cause systemic infection in neonates, and the virus may also cause other clinical disorders including abortion, infertility, conjunctivitis, encephalomyelitis, mastitis, enteritis and lesions in the interdigital space (Straub 2001).

The etiology was shown to be a filterable agent of viral aetiology (Reisinger and Reimann 1928, (Muylkens et al. 2007). The virus was isolated in 1956 (Madin et al 1956) and identified as a bovine herpes virus in 1961 (Armstrong et al. 1961, cf. Yates 1982) and shown to be the same virus group causing IBR, IPV and IBP. There are three main subtypes of BoHV-1 (Miller et al. 1991). Subtype BoHV-1.1 primarily causes IBR with subclinical or clinical infection of the respiratory organs, and subtype BoHV-1.2a may cause IBR and abortion. Subtype BoHV-1.2b may cause respiratory disease but mainly causes infection of the reproductive organs leading to IPV and abortion in females and IBP in males (Edwards et al. 1990, Wentink et al 1993, Graham 2013). However, Nylin (2000) wrote: "As found by others (Mc Kercher et al 1959, Gillespie et al. 1959, Bowling et al. 1969) there was no correlation between the genome type and the clinical manifestations, confirming that a distinction between groups of strains associated with genital and respiratory disease (i.e. IBR/IPV) could not be verified. This was also concluded in the study of Danish BHV-1 strains by Christensen et al. (1996)".

IBR has been diagnosed in cattle (dairy as well as beef) worldwide and only few regions and countries have cleared the infection, i.e. Denmark Finland, Sweden, Switzerland, Austria, Norway, most federal states of Germany and Bolzano in Italy (Straub 1975, 2001; Raaperi et al. 2014). IBR induce disease, reduce welfare and fertility and milk yield and weight gain and impose trading restrictions. This leads to significant economic losses.

IBR is currently the predominating form of disease from infection with BoHV-1 and the present review focuses on IBR only.

A3. Methods

A3.1. Clinical signs, severity, duration, mortality/case-fatality and impact on animal welfare of the individual

A systematic review was carried out in PubMed using the search terms (IBR or infectious bovine rhinotracheitis) and ((clinical sign*) or (clinical disease) or death or mortality) and (cattle or cow or

bovine). The titles and /or abstracts were then reviewed by one reviewer. Review papers were also included and used for snowballing. Exclusion criteria for the systematic review were language (non-English or non-German), not cattle, not on IBR as a comorbidity and experimental studies - e.g. vaccine trials. Information on the clinical signs, their duration and severity was then summarized and tabulated.

A3.2. Within- and between-herd prevalence and incidence in Denmark and selected countries

Prevalence estimates in populations before the onset of control programs were extracted from research papers. Prevalence estimates after implementation of control programs were based on recent publications or from web-pages describing the control programs and their effect in the individual countries.

A4. Results

A4.1. Clinical signs, severity, duration, mortality/case-fatality and impact on animal welfare of the individual

A total of 240 articles were identified in the search on 1st April 2019. After assessment of titles or abstracts and inclusion of articles identified by snowballing a total of 53 publications were included in the review.

Schroeder and Moys (1954) were the first to describe the index case in October 1953 in California and further outbreaks in 52 dairy herds with the clinical appearance of what was later believed to be IBR. In lactating cows the first evidence of infection was a sudden drop in lactation lasting 1-2 days, and the disease spread within the individual herds during 2-6 weeks. Temperatures varied from 40-41 ^{o}C (normal: cattle >1 year 38.0-39.0 ^{o}C; cattle 4-12 months 38.5-40.0 ^{o}C; calves <4 months 38.5-40.5 ^{o}C). Half of the affected animals ceased rumination and refused to eat, and cows seemed constipated shortly before the onset of visible symptoms, while appetite of the others remained until visible respiratory symptoms. Excessive salivation and grinding of the teeth was observed. The mucous membrane of the nostrils was inflamed and with mucopurulent discharge. Respiration was shallow and rapid 30-60 per minute (normal 10-30 per min) and a short explosive cough was characteristic. Pain was not evident on tactile percussion, and auscultation revealed no abnormal sounds in the early stages. Diarrhea was not observed except for dying animals. There was a considerable variation in severity and duration of clinical manifestations among the animals. Of 13,108 cattle in 52 affected herds 1002 became infected (morbidity risk = 7.6 %) and 30 died (case fatality = 3 %). Miller (1955) reported similar clinical findings among beef and dairy cattle in Colorado in young (down to 3 weeks old) as well as matured cattle. The earliest cases were seen in 1950.

Ross et al. (1983) described a case of acute and fatal clinical disease in 7 neonatal calves due to infection with IBR virus. The calves were born from 7 uninfected pregnant replacement heifers newly purchased from a single farm. The calves were fed pooled colostrum from the purchasing farm. All calves developed bilateral serous ocular and nasal discharges, excessive salivation and

pyrexia (40 ^{o}C). Five calves died within 2-3 days due to aspiration pneumonia caused by severe laryngeal edema and difficulty in swallowing milk fed from a bucket. The other two calves recovered slowly over 2 weeks. Thus, the morbidity risk was 100 % and the case fatality was 5/7=71.4 %.

Wiseman et al. (1978) described an outbreak in Scotland of acute IBR in a group of 46 weaned single-suckled calves 6 days after arrival form market. The clinical signs were slight dullness, unwillingness to eat, profuse lachrymation and conjunctivitis, purulent nasal discharge, grooling saliva, pyrexia (39.5-40.5 ^{o}C) and frequent coughing. Morbidity was almost 100 %. One calf would probably have died from the infection, but was euthanized for further investigation, i.e. mortality and case fatality ≈ 2 %. All other calves had recovered over 26 days.

Wiseman et al (1980) described the major clinical findings in 15 cattle herds (10 beef herds, 4 dairy herds and 1 suckler beef cow herd) with outbreaks of IBR in northern Britain (mainly Scotland). The 15 farmers' reasons for seeking veterinary advice varied, and the order (from most to least) of the major reasons were signs of reduced appetite (60 %), serous ocular and nasal discharge (53 %), coughing (47 %), dullness(40 %), tachypnea (33 %), drooling saliva (27 %), nasal discharge (20 %), ocular discharge (13 %), hyperpnoea (13 %), reduced milk yield (13 %), and blood on the tail (7 %). Clinical signs of IBR developed within four weeks upon arrival of animals from a market in 14 of the 15 herds. The within herd morbidity risk varied from ca. 10 % in one herd to >90 % in 10 of the 15 herds. The within herd mortality risk varied from zero to 8 %. The clinical examinations showed the following signs with varying extent in all outbreaks: Pyrexia often at 41 ^{o}C, dullness, reduced appetite, bilateral nasal discharge (serous to mucoid or purulent), and increased frequency of coughing, tachypnea and hyperpnoea and ocular discharge. Reduced body weight was in severe cases up to 30 kg and beef cattle failed to maintain their previous rate of weight gain for a period of 3-5 weeks. Milk yield was decreased from low up to 24 liters per day usually for a few days and in severe cases sometimes they became agalactic. Abortion was seen with or without prior clinical signs. Clinical signs in the infected herds were observed for one to eight weeks. In general the disease took 3-5 weeks to pass through a herd (Wiseman et al. 1980).

Pritchard et al. (2003) described a single dairy herd outbreak of IBR. After the herd had tested serologically free from IBR for 13 years on bulk milk, it was detected weakly positive apparently without any clinical signs in infected cows. Blood testing in May 2000 of all 231 cows showed 70.1 % cows to be seropositive for IBR virus, and none of the heifers tested seropositive. A follow up interview with the herdsman, however, revealed that there 1-2 months previously had been a slight bilateral watery ocular discharge from ca. 5 % of the cows without any other clinical signs before or after the testing. Two cows that aborted in year 1999 and 4 cows in year 2000 all tested positive for IBR. Thus, clinical signs can be so weak that they pass without notice and action from the farmer.

Table A1. Clinical manifestations and disease stages of IBR infections, duration and implications for animal welfare at animal level.

**Clinical manifestation**	**Typical signs**	**Frequency of clinical signs. (Morbidity)**	**Duration**	**Welfare implications**	**Remarks**
Overall assessment of acute phase signs	Overall assessment (see details below)	Morbidity 100 %<br>Mortality <10 %	One week. All evidence of the acute phase disappear in less than one mth.	Generally severe	Most clinical signs varies considerably among cases
Detailed signs during the acute phase	Milk yield reduced 4-24 liters/cow day, or agalactic	100 %<br>High	Few days	None	
	Pyrexia (40-41°C)	100 %<br>High	Several days	Severe	
	Reduced appetite and rumination.<br>Slight-anorexic and constipated	High	Several days - 3-5 weeks	Low	
	Salivation slight to excessive (drooling)	High	Several days	Low	
	Nasal discharge serous-mucoid-mucopurulent	High	5-7 days	Low	
	Respiration shallow and rapid (30-60/min)<br>Dyspnea	High	Several days	Very severe	
	Short coughing varies in strength	Medium - high	Several days	Very severe	

	Dehydration Varies	?	Several days	Low	
	Dullness (severity varies)	100 %	Several days	Low	
	Ocular discharge, lachrymation, conjunctivitis. Severity varies	High	Several days	Low	
	Abortion (primarily during 2nd and 3rd trimesters)	Low	Short time	Severe	
	Transient diarrhoea	Low	Few days	Medium	
	Mortality	<10 %			
Latent sub-clinical phase	None	High	Persistent infection in ganglia	None	There are no reports on the impact of this chronic sub-clinical phase

*Wiseman et al. (1980).

From the literature it can be concluded that there are no pathognomonic signs for IBR. All signs can be seen in connection with many other diseases and are individually not very strong diagnostic indicators. As an example of the weakness of the clinical signs in relation to diagnostic reasoning, nasal swabs were sampled from clinically suspected Dutch farms on four occasions (Anon 2018a, 2018b, 2018c, 2018d, 2019). The positive predictive values for detection of IBR field strains are estimated from these reports in Table A2 and vary between 0.08 and 0.19. This is very low values compared to the generally high within-herd prevalence when the infection is present.

Table A2. Estimation of the positive predictive value of nasal swabs from IBR clinically suspected cattle farms in The Netherlands 2018-2019.

Time period	# farms sampled	# farms positive	PPV
2018 1st quarter	57	11	0.19
2018 2st quarter	61	10	0.16

2018 4th quarter	38	3	0.08
2019 1st quarter	50	6	0.12

A4.2. Within- and between-herd prevalence in Denmark and eight other EU member states, and with specific comparison to The Netherlands, Belgium and Ireland

Table A3 summarizes 12 selected surveys on the occurrence of BHV-1 infections in dairy and beef and mixed cattle herds in Belgium, Estonia, Hungary, Ireland, Italy, Spain, The Netherlands and the United Kingdom. The surveys are based on bulk milk samples and/or individual blood samples from various fractions of the cattle populations and with more or less precise information on the sampling frames and sampling methods. Generally the studies are based on random or close to random selection of herds and animals. All surveys are based on a commercial ELISA antibody test type except the study in Italy by Castrucci (1997) who used the serum neutralization test. Some studies also estimate the test sensitivity and specificity and present apparent and true prevalence estimates. Estimates extracted from the publications mainly concern the unvaccinated cattle population. Generally across the studies the between-herd prevalence varies from 13 % in Hungary to 98.8 % in Italy; the within-herd prevalence is not very often available and the four examples vary from 20.1 % in young stock to 52 % in mature cows; and the individual level prevalence varies between 26.8 % and 77.5 % and is not available in all studies. Dutch dairy farms had during 2015-2016 a between-herd prevalence of 15.6 % with IBR antibodies in bulk milk and in non-dairy farms the between-herd prevalence was 9.6 % (Anon 2018a).

None of the listed countries had an eradication program implemented at the time of study. However, several countries for periods used vaccination to control the disease in parts of their populations.

Table A3. Summary of 12 European surveys of the prevalence of BHV-1 infection in cattle herds.

Country	Study period	Sampling frame	Sampling method		Sample size		Prevalence (%)			Comments	Reference
			Herds	Animals	Herds	Animals	Between-herd	Within-herd	Individual level		
Belgium	1997-1998	All provinces of Belgium	Random stratified within dairy, beef and mixed	All animals within each herd	207	4060	TP=65	TP=34	TP=36	Not vaccinated	Boelaert et al. 2000
Estonia	2006-2008	15 counties in 5 dairy regions in Estonia	Stratified random within 5 dairy regions	Age stratified random within herd	328 herds for BTM samples<br>64 herds for serum samples	9637 serum samples	TP=22.0	37.8<br>Youngstock: 20.1<br>Cows: 52.8		Not vaccinated	Raaperi et al. 2010
Hungary	1992-1993	All small herds <50	All small herds	All animals aged >24 months	63,373	Bulk milk from dairy herds and pooled	13.5			Not vaccinated	Tekes et al. 1999

						serum from beef herds			
Hungary	1992-1993	75 % of large herds≥50	Considere d random	All animals aged >24 months	736	70,525 pooled samples each of <10cattle<br>Milk from lactating cows, serum from beef and dry cows	79.3		Not vaccinated	Tekes et al. 1999
Hungary	1997	90 % of small herds <50	Considered random	All animals aged > 24 months	45,899	Serum samples pooled from <10 animals per herd	15.7		Not vaccinated	Tekes et al. 1999
Ireland	2007	Beef herds sending bulls to performance testing sta-	Purposive	All	41	Ca. 1125 serum samples from candidate	73.2	28	Not vaccinated	O’Grady et al. 2008

		tions				bulls, dams and cohort animals						
Ireland	2009	Four provinces. Dairy and beef herds	Random stratified for dairy and beef		781 beef herds and 394 dairy herds	One serum pool from each herd based on ≤30 animals	74.9				Not vaccinated	Cowley et al. 2011
Italy	Ca. 1996	Region of Lombardia (other regions in the study ignored)	Considered random among herds with no history of recent vaccination	Not mentioned	51	6,415	84.3			35.0	Not vaccinated recently	Castrucci et al. 1997
Italy	Ca. 2004	Provinces Campania, Puglia, Basilicata	Stratified random	Age stratified random within herd	81	948 blood samples	98.8			77.5	Not vaccinated	Rinaldi et al. 2007

Spain	2000	Andalucia	Random	Random within herd	110	2393, ca. 25 blood samples from each herd	TP=70.4		TP=45.7	Not vaccinated. Represents small and medium sized dairy and beef herds	Gonzales-Garcia et al. 2009
Spain	2000	Galicia	Stratified random	All cattle aged > 1 year	375 dairy<br>385 beef	Individual serum from 6,038 dairy<br>2,690 beef	TP=58.3<br>TP=48.1		TP=43.2<br>TP=26.8	Some herds vaccinate	Eiras et al. 2009
Spain	2004	Galicia	Stratified random	All cattle aged > 1 year	1,147 dairy<br>1,464 beef	Individual serum from 38,120 dairy<br>21,219 beef	TP=51.5<br>TP=45.2		TP=37.8<br>TP=33.1	ADSG* herds	Eiras et al. 2009
The Netherlands	1994	The whole country	All Ducth dairy		33,636 dairy	None	84.0			Some vaccinate.	Van

			herds		herds					Status unknown	Wuijckhuise et al. 1998
United Kingdom	2002-2004	South West England. Farms from the Randomised Badger culling Trial and farms depopulated in the 2001 FMDepidemic	Purposive	All accessible cattle > 2 years	107	14,243 animals (serum samples)	83.2	43.1	42.5	Not vaccinated	Woodbine et al. 2009

* Dairy and beef herds enrolled in a voluntary health defence program restricted to only marker vaccines, sanitary measures and control programs for cattle trade, disinfection, decontamination and BVD, Johne’s disease and neosporosis.

IBR-infection in Danish cattle was diagnosed for the first time in January 1969 in semen and preputial secretion from a bull at an artificial insemination center (Bitsch 1973), and a national eradication program in Denmark was initiated by September 1984 (Nylin 2000). In the beginning of 1984 before start of the eradication program, the apparent prevalence of positive dairy herds (i.e. the between-herd prevalence) was 9 %. The prevalence reached maximum at 11 % in 1985 in the early phase of the eradication program. Figure B1 shows the number of BoHV-1-infected dairy herds monthly during the eradication campaign, and Denmark obtained the EU official BHV-1 free status in December 1992 (Nylin et al. 1998, Commission Decision 2004/558/EEC). Unfortunately it is not uncommon to have re-occurrence of disease after eradication, and during the period 1993-1996, 2000, 2002, 2003 and 2005 sporadic outbreaks were seen. Table A4 presents a summary of the above prevalence estimates. However, Denmark maintained the EU official BHV-1 free status.

Reintroduction of BoHV-1 infection in the Danish cattle population in 1995 comprised 61 dairy herds identified in the surveillance program with tests of live animals and at slaughter. Clinical signs observed in 6 herds with confirmed virus isolation in 1995 were characterized in interviews with the veterinary practitioner as follows (Nylin et al. 1998): Mild (only discharge from eyes and nose), severe (dyspnoe and fever) and very severe (restricted respiration, dyspnoe and pulmonary oedema resulting in death of the animal). Two herds showed only mild signs, three herds showed severe signs and one herd showed very severe signs.

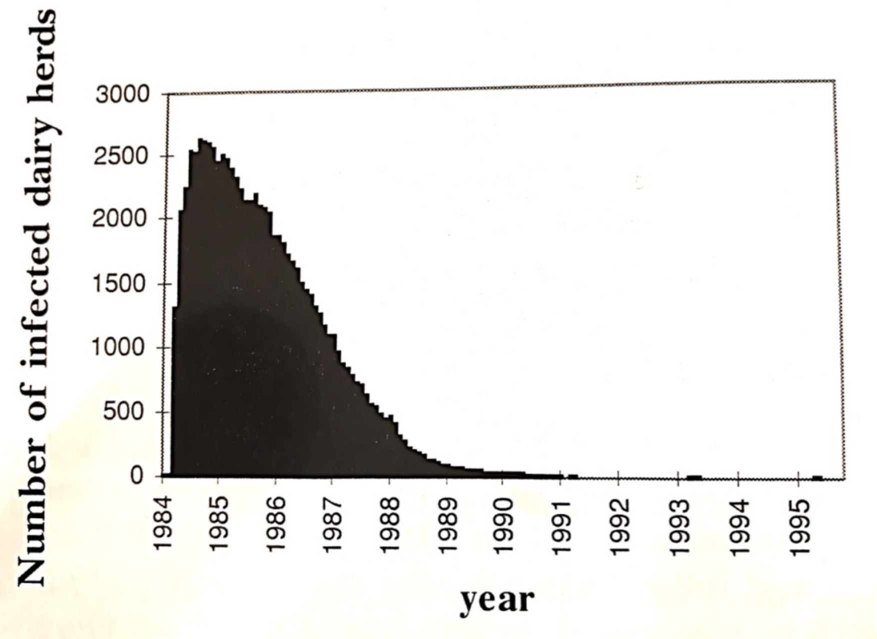

Figure B1. The number of registered BHV-1 infected dairy herds in Denmark in the period 1984-1995. Numbers were based on extract from the IBR-register. Copied from Nylin (2000, page 32).

Table A4. Summary table of typical occurrence of IBR infections in endemic areas with no control program.

Infection stage	Population level prevalence			
	Individual level	Mortality	Within-herd	Between-herd
Antibody positive	10-78 %	<10 %	20-52 % (increases with herd size)	9- 99 %

A5. References

Ackermann M, Engels M 2006. Pro and contra IBR-eradication. Vet Microbiol 113, 293-302.

Ackermann M, Wyler R 1984. The DNA of an IPV strain of bovid herpesvirus 1 in sacral ganglia during latency after intravaginal infection. Vet Microbiol 9, 53-63.

Animal Health Ireland 2015. National perspectives on IBR. Control and eradication in Belgium and The Netherlands. Report of the infectious bovine rhinotracheitis (IBR) Technical Working Group (TGW) of Animal Health Ireland on a study visit to Belgium and the Netherlands 2015. http://animalhealthireland.ie/wp-content/uploads/2016/05/IBR-Study-Visit-Web.pdf

Anonymous 2018a. Monitoring Animal Health, Cattle. Highlights Report, First Quarter 2018. GD Animal Health, Deventer, The Netherlands. file:///C:/Users/cwd117/Downloads/Highlights%20Report%20Cattle%20First%20Quarter%202018.pdf (visited 31 October 2019)

Anonymous 2018b. Monitoring Animal Health, Cattle. Highlights Report, Second Quarter 2018. GD Animal Health, Deventer, The Netherlands. file:///C:/Users/cwd117/Downloads/Highlights%20Report%20Cattle%20Second%20Quarter%202018.pdf (visited 31 October 2019)

Anonymous 2018c. Monitoring Animal Health, Cattle. Highlights Report, Third Quarter 2018. GD Animal Health, Deventer, The Netherlands. file:///C:/Users/cwd117/Downloads/Highlights%20cattle%20third%20quarter%202018%20(1).pdf (visited 31 October 2019)

Anonymous 2018d. Monitoring Animal Health, Cattle. Highlights Report, Fourth Quarter 2018. GD Animal Health, Deventer, The Netherlands. file:///C:/Users/cwd117/Downloads/Cattle%20Health%20Surveillance%202018-4%20(1).pdf (visited 31 October 2019)

Anonymous 2019. Monitoring Animal Health, Cattle. Highlights Report, First Quarter 2019. GD Animal Health, Deventer, The Netherlands. file:///C:/Users/cwd117/Downloads/Cattle%20Health%20Surveillance%202019-1.pdf (visited 31 October 2019).

Armstrong JA, Pereira HG, Andrewes CH 1961. Observations on the virus of infectious bovine rhinotracheitis and its affinity with the herpesvirus group. Virology 1961, 14, 276-285.

BEK nr. 144 af 03/04 1984. Bekendtgørelse om IBR/IPV-infektioner hos kvæg. (Danish order on IBR/IPV infections in cattle). Landbrugsministeriet.

BEK nr. 1332 af 30/11 2017. Bekentgørelse om IBR-infektion. Miljø- og Fødevareministeriet. (Danish order on IBR-infection in cattle).

Bitsch V 1973. Infectious bovine rhintracheitis virusinfectn in bulls, with secial reference to preputial infectin. Applied Microbiol 26, 337-34.

Boelaert F, Biront P, Soumare B, Dispas M, Vanopdenbosch E, Vermeersch JP, Raskin A, Dufey J, Berkvens D, Kerkhofs P 2000. Prevalence of bovine herpesvirus-1 in the Belgian cattle population. Prev Vet Med 45, 285-295.

Bowling CP, Goodheart CR, Plummer G 1969. Oral and genital bovine herpesviruses. J Virol 3, 95-97.

Castrucci G, Martin WB, Frigeri F, Ferrari M, Salvatori D, Tagliati S, Cuteri V 1997. A serological survey of bovine herpesvirus-1 infection in selected dairy herds in Northern and Central Italy. Comp. Immun Microbiol infect Dis 20(4), 315-317.

Christensen LS, Madsen KG, Nylin B, Rønsholt L 1996. A contribution to the systematization of bovine herpesvirus 1 based on genomic mapping by restriction fragment pattern analysis. Virus Res 46, 177-182.

Commission Delegated Regulation 2018/1629 2018. Commission Delegated Regulation (EU) 2018/1629 of 25 July 2018 amending the list of diseases set out in Annex II to Regulation (EU) 2016/429 of the European Parliament and of the Council on transmissible animal diseases and amending and repealing certain acts in the area of animal health ('Animal Health Law') https://eur-lex.europa.eu/legal-content/EN/TXT/PDF/?uri=CELEX:32018R1629&from=EN

Council Directive 88/407/EEC 1988. Council Directive of 14 June 1988 laying down the animal health requirements applicable to intra-Community trade in and imports of deep-frozen semen of domestic animals of the bovine species (88 / 407 /EEC)

Cowley DJ, Clegg TA, Doherty ML, More SJ 2011. Aspects of bovine herpesvirus-1 infection in dairy and beef herds in the Republic of Ireland. Acta Vet Scand 53, 40, 1-9.

Danish Veterinary and Food Administration 2018. Animal Health in Denmark 2017. Ministry of Environment and Food of Denmark. September 2018. Publication number 2018206. 84 pp.

Edwards S, White H, Nixon P 1990. A study of the predominant genotypes of bovid herpesvirus 1 found in the U.K. Vet. Microbiol 22, 213-223.

Eiras C, Dièguez FJ, Sanjuán ML, Yus E, Arnaiz I 2009. Prevalence of serum antibodies to bovine herpesvirus-1 in cattle in Galicia (NW Spain). Spanish J Agric Res 7(4), 800-806.

Federal Agency for the Safety of the Food Chain 2015. Circular letter concerning the additional conditions of trade in bovine animals to and from Member States or regions having the Article 9 or Article 10 status as regards IBR. Reference number PCCB/S2/LVH/1258456. http://www.afsca.be/dierlijkeproductie/dieren/omzendbrieven/_documents/2015-02-26CIRCULAR_LETTRE_IBR_ART_9_STATUS_BE.pdf

Gillespie JH, McEntee K, Kendrick JW, Wagner WC 1959. Comparison of infectious pustular vulvo-vaginitis virus with infectious bovine rhinotracheitis virus. Cornell Vet 49, 288-297.

Gonzales-Garcia MA, Arenas-Casas A, Carbonero-Martinez A, Borge-Rodriguez C, Garcia-Bocanegra I, Maldonado JL, Gomez-Pacheco JM, Perea-Remujo JA 2009. Seroprevalence and risk factors associated with bovine herpesvirus type 1 (BHV1) infection in non-vaccinated cattle herds in Andalusia (South Spain). Spanish J Agric Res 7(3), 550-554.

Graham DA 2013. Bovine herpes virus-1 (BoHV-1) in cattle - a review with emphasis on reproductive impacts and the emergence of infection in Ireland and the United Kingdom. Ir Vet J. 2013, 66(1):1-15. doi: 10.1186/2046-0481-66-15.

Madin SH, York CJ, Mckercher DG 1956. Isolation of IBR virus. Science 124, 721-722.

MC Kercher DG, Straub OL, Saito JK, Wada EM 1959. Comparative study of the etiologic agents of infectious bovine rhinotracheitis and infectious pustular vulvovaginitis. Can J Comp Med 23, 320-328.

Miller NJ 1955. Infectious necrotic rhinotracheitis of cattle. J Amer Vet MedAssoc 126, 463-467.

Miller JM, Whetstone CA, van der Maaten MJ 1991. Abortifacient property of bovine herpesvirus type 1 isolates that represent three subtypes determined by restriction endonuclease analysis of viral DNA. Amer J Vet Res 52, 458-461.

More S et al 2017. Assessment of listing and categorization of animal diseases within the framework of the Animal Health Law (Regulation (EU) No 2016/429: indectious bovine rhinotracheitis (IBR). EFSA Journal 2017, 15 (7), 4947. 25 pages.

Muylkens B, Thiry J, Kirten P, Schynts P, Thiry E 2007. Bovine herpes virus 1 infection and infectious bovine rhinotracheitis. Vet Res, 38, 181-209.

Nylin B, Madsen KG, Rønsholt L 1998. Reintroduction of Bovine Herpes Virus Type 1 into Danish cattle herds during the period 1991-1995: A review of the investigations in the infected herds. Acta vet. Scand. 39, 401-413.

Nylin B 2000. Bovine Herpes Virus-1 (BHV-1) in Danish Cattle Herds. A retrospective evaluation of the diagnostic methods used in the BHV-1 eradication campaign, and an initial study of the effect of the herd size on the BHV-1 infection status. The Royal Veterinary and Agricultural University, Department of Animal Science and Animal Health, Division of Ethology and Health, Frederiksberg, Denmark. PhD. Thesis.136 pp.

O'Grady L, O-Neill R, Collins DM, Clegg TA, More SJ 2008. Herd and within-herd BoHV-1 prevalence among Irish beef herds submitting bulls for entry to a performance testing station. Irish Vet J 61 (12), 809-815.

Pritchard GC, Banks M, Vernon RE 2003. Subclinical breakdown with infectious bovine rhiotracheitis virus infection in dairy herd of high health status. Vet Rec 153, 113-117.

Raaperi K, Nurmoja I, Orro T, Viltrop A 2010. Seroepidemiology of bovine herpesvirus 1 (BHV1) infection among Estonian dairy herds and risk factors for the spread within herds. Prev Vet Med 96, 74-81.

Raaperi K, Orro T, Viltrop A 2014. Epidemiology and control of bovine herpesvirus 1 infectoin in Europe. The Vet Journal 201, 249-256.

Reisinger L, Reimann H 1928. Beitrag zur Ätiologie des Bläschenausschlages des Rindes. Wien tierärztl. Mschr. 15, 249-261.

Rinaldi L, Pacelli F, Iovane G, Pagnini U, Veneziano V, Fusco G, Cringoli G 2007. Survey of Neospora caninum and bovine herpes virus 1 coinfection in cattle. Parasitol Res 100, 359-364.

Ross HM, Hunter AR, Masson AG, Nettleton PF 1983. Fatal infection of neonatal calves by infectious bovine rhinotracheitis virus. Vet Rec 113, 217-218.

Schroeder RJ, Moys MD 1954. An acute upper respiratory infection of dairy cattle. JAVMA 1954, 125, 471-472.

Straub OC 1975. Infectious bovine rhinotracheitis virus. History and recent developments. Dev Biol Stand. 28, 530-533.

Straub OC 2001. Advances in BHV1 (IBR) research. Deutsche Tierarztlighe Wochenschrift 108, 419-422.

Tekes L, Markos B, Kecskemétt S, Méhesfalvi J, Máté Z, Kudron E 1999. Prevalence of bovine herpesvirus 1 (BHV-1) infection in Hungarian cattle herds. Acta Vet Hungarica 47, 303-309.

Tousimis AJ, Howells WV, Griffin TP, Porter RP, Cheatham WJ, Maurer FD 1958. Biophysical characterization of infectious bovine rhinotracheitis virus. Proc Soc Exp Biol Med 1958, 99, 614 - 617.

Van Wuijckhuise L, Bosch J, Franken P, Frankena K, Elbers ARW 1998. Epidemiological characteristics of bovine herpesvirus 1 infections determined by bulk milk testing of all Dutch dairy herds. The Vet Rec 142, 181-184.

Wentink GH, van Oirschot JT, Verhoeff J 1993. Risk of infection with bovine herpes virus 1 (BHV1): A review. Vet Quarterly 15, 30-33.

Winkler MT, Doster A, Jones C 2000. Persistence and reactivation of bovine herpesvirus 1 in the tonsils of latently infected calves. Journal of Virology 74, 5337-5346.

Wiseman A, Msolla PM, Selman IE, Allan EM, Cornwell HJC, Pirie HM, Imray WS 1978. An acute severe outbreak of infectious bovine rhinotracheitis: Clinical, epidemiological, microbiological and pathological aspects. Vet Rec 103, 391-397.

Wiseman A, Msolla PM, Selman IE, Allan EM, Pirie HM 1980. Clinical and epidemiological features of 15 incidents of severe infectious bovine rhinotracheitis. Vet Rec 107, 436-441.

Woodbine KA, Medley GF, Moore SJ, Ramirez-Villaescusa AM, Mason S, Green LE 2009. A four year longitudinal sero-epidemiological study of bovine herpesvirus type-1 (BHV-1) in adult cattle in 107 unvaccinated herds in south west England. BMC Vet Res Jan 30; 5:5. Doi: 10.1186/1746-6148-5-5.

Yates WD 1982. A review of infectious bovine rhinotracheitis, shipping fever pneumonia and viral-bacterial synergism in respiratory diseases of cattle. Can. J. Comp Med 46, 225-263.

B. Paratuberculosis in cattle - systematic search and review of clinical manifestations.

By Søren Saxmose Nielsen

B1. Summary

Paratuberculosis in cattle - systematic search and review of clinical manifestations.

Paratuberculosis is a chronic infection of cattle and other ruminants, caused by *Mycobacterium avium* subsp. *paratuberculosis* (MAP). The infection can after a prolonged latency period without clinical disease cause slow emaciation and recurrent diarrhoea, which at a later stage can lead to persistent diarrhoea and ultimate death.

We made a systematic literature compilation on the clinical signs of paratuberculosis. Then, we made a qualitative comparison of efforts against paratuberculosis in four European countries: Sweden, Austria, the Netherlands and Denmark. Sweden was chosen because it is believed that the country has a low incidence and it is legally required that paratuberculosis is eradicated following any suspicion. Austria was chosen because clinical suspicions must be reported, but paratuberculosis is not necessarily eradicated from the herd of origin and can thus spread further. The Netherlands was chosen because of the focus on milk quality, not spread of infection and animal welfare. Denmark focuses on reducing the prevalence of infection, which affects both the spread of infection, production and clinical disease.

Infections with MAP can be divided into subclinical and clinical stages. In the clinical stages, emaciation may be observed following recurrent diarrhoea, which after shorter (days) or longer (typically months) progresses to persistent diarrhoea, total emaciation and death.

More than half of the Danish, Austrian and Dutch dairy herds are estimated to be infected with MAP, but the prevalence at cow level has only been estimated in Denmark, where in 2015 it was estimated to be approx. 6%. This figure is associated with considerable uncertainty, but there was a significant decrease in the prevalence in the period from the introduction of the voluntary Danish control programme in 2006 until the study in 2015. No studies have been conducted on the occurrence of clinical disease in the countries included.

B2. Introduction of the disease

Paratuberculosis, or Johne's disease, is a chronic infection of cattle and other ruminants caused by *Mycobacterium avium* subsp. *paratuberculosis* (MAP). Infection is often divided in four stages: I) silent infection; II) subclinical disease; III) clinical disease; and IV) advanced clinical disease (Whitlock and Buergelt, 1996). MAP infections may affect production such as reduced milk yield (Kudahl et al., 2004) and slaughter weight (Kudahl and Nielsen, 2009) in Stages II-IV, but premature culling or death can also occur if the disease progresses. Furthermore, MAP has been associated to Crohn's disease in humans, where treatment with anti-mycobacterial drugs has shown an effect in a few randomized clinical trials. Live MAP has also been recovered from retail milk. However, the role of MAP in Crohn's disease remains to be established (More et al., 2017).

Disease control programmes have been implemented in many countries globally, often with very different purpose due to the different effects. The chronic nature of the infection and disease has a number of implications for the approach to establishment of disease control programmes. Furthermore, data collection in relation to the programmes is often of a diverse nature. Firstly, many scientific studies suffer from selection bias, e.g. because animals are lost to follow-up. Spectrum bias is also common because the most diseased animals are easier to confirm as diseased. Secondly, misclassification bias is common because of the protracted course of the infection, and because the same test does not fit all purposes that may be relevant in the disease control programmes (Nielsen, 2014). Animal welfare has not specifically been studied in relation to MAP infections. Death or culling due to clinical disease may serve as the best proxies for animal welfare, although reported culling due to other MAP related diagnostic evidence is also the basis for decisions on culling.

The overall aim of this work was to assess the available information on the impact of MAP infection on animal. The objectives of this report were therefore to summarise a) the clinical signs, their severity and duration; and b) the mortality and case-fatality of MAP infections based on a systematic literature review.

B3. Methods

B3.1. Clinical signs, severity, duration, and mortality/case-fatality

A systematized review (Grant and Booth, 2009) was carried out in PubMed using the search terms: (paratuberculosis or Johne's disease) and ((clinical sign*) or (clinical disease) or death or mortality) and (cattle or cow or bovine). The abstracts were then reviewed as follows: one reviewer assessed all the abstracts and included papers with: original descriptions/recordings of clinical disease (with aspects of mortality, diarrhea, emaciation, pain, bottle jaw, etc. but not milk yield). Review papers were also included and used for snowballing (Greenhalgh and Peacock, 2005). Exclusion criteria for the systematic review were: a) language: non-English or non-German; b) not on paratuberculosis or paratuberculosis as acomorbidity; c) not cattle; d) not original study of clinical disease. Information on the clinical signs, their duration and severity was then summarised and tabulated.

B3.2. Within- and between-herd prevalences /incidences in Denmark, Sweden, the Netherlands and Austria

Recent information on the prevalence of MAP infections in Denmark, Sweden, the Netherlands and Austria was included. Sweden was included because of their proclaimed low prevalence of MAP infections, the Netherlands was included because they have a long-standing milk quality control programme, which differs from the programmes in many other countries, and Austria was selected because clinical MAP infections are notifiable in Austria. Prevalence estimates were based on a systematic review from 2007 (Nielsen and Toft, 2009) updated with information from the peer-reviewed literature and information published from the international ParaTB Forum. Prevalence estimates before and after intervention was included when possible.

B4. Results

B4.1. Clinical signs, severity, duration, and mortality/case-fatality

A total of 453 hits were identified at the search on 7 March 2019. Of these, 386 could be assessed based on the Abstract, whereas full papers had to be retrieved to assess the remainder. Studies were excluded for the following reasons: a) two studies were in Polish or in Dutch; b) 11 studies were excluded because they were not on paratuberculosis or paratuberculosis was a comorbidity; c) 63 were not on cattle; d) 252 were excluded because no clinical information was included, and an additional 104 papers were excluded for not including specific clinical information. Four reviews were included for snowballing. This resulted in 17 papers with clinical information. An additional eight papers were included as a result of snowballing.

Reported clinical manifestations included a variety of the signs: chronic wasting, scouring, emaciation, intermittent diarrhoea. A number of other signs were also mentioned in literature, but these were not described as part of the recorded data in the studies.

Animals with diarrhoea are considered to be in a catabolic state, while animals without diarrhoea are considered to be in an anabolic state. Protein leakage and impaired amino acid uptake results in a negative nitrogen balance with chronic wasting and emaciation (Chiodini et al., 1984). However, absence of diarrhoea can also be associated with chronic wasting and emaciation. For example, Patterson et al. (1965) reported on six cows with clinical signs: all had severe loss of condition, additionally three experienced diarrhoea (one with severe diarrhoea), two had loose faeces and one had firm faeces. Patterson et al. (1968) also reported on 24 cattle with suspicion of clinical disease. Seventeen of these developed terminal disease characterised overall by prostration, inappetence and extreme muscular weakness, where after they died in 1-2 days. Most of the animals were included in the study due to emaciation, but diarrhoea was only persistent in the terminal stages. Van Amstel (1984) reported on two animals with clinical signs of severe loss of condition (MAP infection confirmed by thickened and corrugated ileum); only one of the cows presented with diarrhoea, whereas the others did not develop diarrhoea over the three week period of hospitalization, where the animal was observed. Prewett (1950) also described multiple cows with clinical signs; diarrhoea and emaciation; diarrhoea and poor condition; emaciation; intermittent diarrhoea. Some of the cows had positive, others a negative bacterial culture. Mortier et al. (2015) recorded development of clinical disease in two of five experimentally infected calves. One had deteriorating body condition from 11.5 months after inoculation, where the body condition score was 2.5, and at 14 months of age, the BCS was 2. Within two weeks, diarrhoea presented intermittently until euthanasia at 16 months post infection. The second calf suffered from acute disease with severe abdominal pain, lack of appetite and diarrhoea at 16 months post infection. Disease deteriorated and the calf was euthanized seven days after onset of the clinical symptoms. Both calves were MAP antibody-positive from 5 months of age. Further to this, Gonzalez et al. (2005) reported clinical disease in 19/47 cows, of which the selection was unclear. All of these 19 were classified based on weight loss and some with emaciation and muscle atrophy. Seventeen had diarrhoea. All 19 had histopathological lesions consistent with MAP infection. Of the remaining 28

without clinical disease, 15 also had histopathological lesions consistent with MAP infection. Khol et al. (2012) selected cows from three herds based on a suspicion of MAP infection, and noted that seven of 58 cows had clinical disease (diarrhoea and weight loss). However, the selection criteria were obscure and the distribution of disease is not necessarily informative. A summary of the clinical information is given in Table B1.

Table B1. Clinical manifestations/disease stages of *Mycobacterium avium* subsp. *paratuberculosis* infections, duration and implications for animal welfare. Information was retrieved from Whitlock and Buergelt (1996) and Brady et al. (2008), but no specific data summaries can be identified in literature. When specific references are given, additional information was retrieved from that reference (BCS= Body condition score). Stages I and II are subclinical and therefore not included.

Disease stage	Typical signs	Frequency of signs	Duration	Welfare implications
Stage III or IV	Weight loss/ Poor condition (BCS 1-2)	1/10 infected animals	3-6 months	Low
	Chronic wasting	1/10 infected animals	3-6 months	Moderate
	Intermittent diarrhoea	1/10 infected animals	3-6 months	Moderate
Stage IV	Emaciation (BCS 0-1)	1/20 infected animals	Days to weeks	Severe
	Pipe stream diarrhoea	1/20 infected animals	Days to weeks	Severe
	Intermandibular oedema	1/20 infected animals	Days to weeks	Severe
	Lethargic	1/20 infected animals	Days to weeks	Severe
	Death	1/20 infected animals	Days to weeks	None

To conclude, few studies provide systematic information on the clinical signs and their duration. The information that was extractable suggests that poor condition is the first visible sign, and this may lead to emaciation. Intermittent diarrhoea can be present, but not in all animals. Eventually, most cattle die from the infection, if they are not culled or euthanized. Very limited primary information is available on the duration. Several reviews listed the duration of Stage III to 3-6

months and Stage IV from days to weeks, but the period from Stage II to Stage IV can be weeks (Whitlock and Buergelt, 1996). Only the study by Mortier et al. (2015) presents primary data to support this information.

Concerning the extent of clinically diseased cattle, there is limited specific information from a random selection of infected herds. However, from selected herds, Larsen and Merkal (1968) reported an annual clinical disease incidence of 4.2% in a herd with reported poor hygiene and an annual clinical incidence risk of 1.4% in a herd with good hygiene. Raizman et al. (2007) reported that 60% and 40% of cull cattle were due to clinical Johne's disease in two large American dairy herds, and Shahmoradi et al. (2008) reported within-herd clinical prevalences in the range 1 to 16%, with a median within-herd prevalence of 2%. Paolicchi et al. (2013) reported a clinical prevalence of 2/77, where both animals presented with diarrhoea and emaciation. In a Norwegian herd, Holstad et al. (2005) reported no clinical disease in a Norwegian herd of 45 animals with eight sero-reactors and confirmed MAP in the herd and histopathological lesions consistent with MAP infection in four animals. Reported prevalences of clinical disease are reported in Table C2 from Minnesota, USA at different stages in a control programme. From the older literature, Reinders (1963) reported within-herd prevalences of clinical disease in the range 0 to 60%, with a median of 17% in thirty herds. Such observations do not appear to be common in today's cattle production. However, they illustrate the point that it is indeed possible to observe high levels of clinical disease.

Table B2. Reported prevalences of clinically diseased cattle due to MAP infections at different stages in control programs.

Period	Case-fatality	Reference
2-1 years before onset of control	3.6% annually	Ferrouillet et al. (2009)
1-0 years before onset of control	1.6% annually	Ferrouillet et al. (2009)
0-1 years from onset of control	2.5% annually	Ferrouillet et al. (2009)
1-2 years after onset of control	0.4% annually	Ferrouillet et al. (2009)
4 years	8.8% over four years in cohort of 260 MAP exposed animals	Espejo et al. (2013)

Overall, the age distribution at first reporting of clinical signs of 171 animals from four herds has been described to minimum age: 6 months; peak: 4 years; mean: 5 years; maximum 13.5 years (Matthews, 1947). A similar distribution was observed by Jubb and Galvin (2004b). The distribution suggests that most cows break down around 4 to 5 years of age, which is supported by data

presented by Reinders (1963), where up to 8.4% of the age-group experienced clinical disease (Figure C1).

The prevalences listed above are all from more recent studies, where the rearing conditions and hygienic level may be significantly better than in the past. The estimates are in contrast to the conditions in an experimental herd with high infection pressure. Here, 7/23 animals born in the herd died of clinical paratuberculosis at an average of 32.3 months of age; an additional 6 animals slaughtered at on average 22.8 months of age had MAP associated lesions at slaughter. The remaining 10 showed no sign of infection after on average 26.2 months of age (Hagan, 1938). A clinical prevalence such as this does not seem likely under modern rearing conditions.

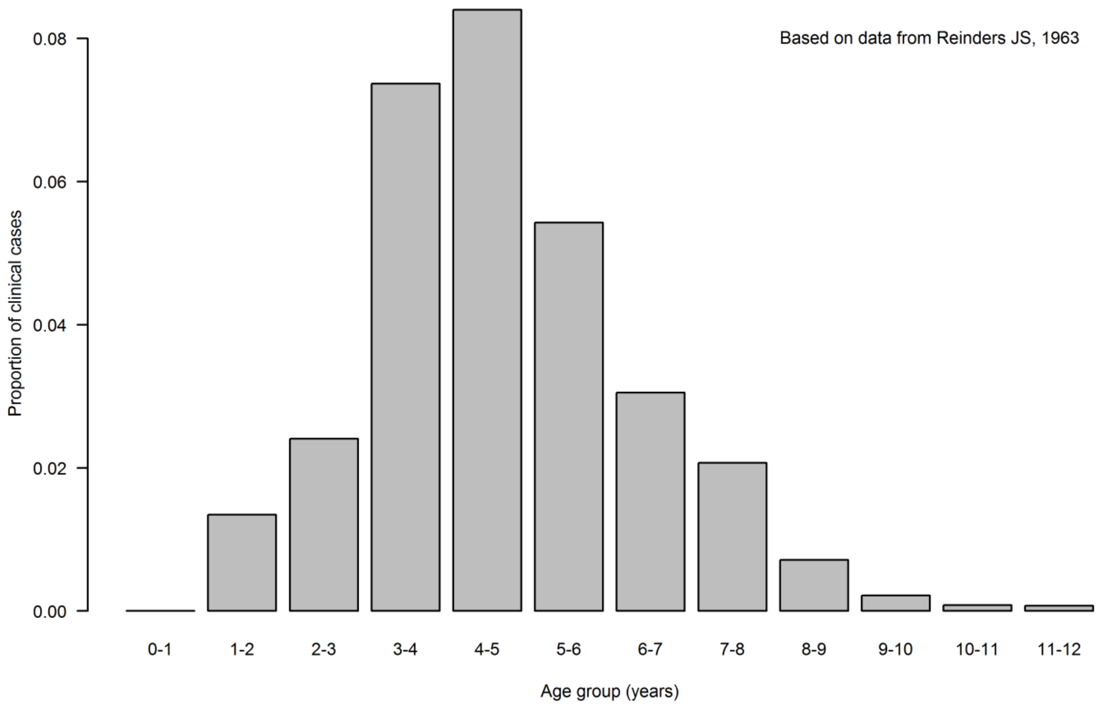

Figure B1. Fraction of age-group with clinical MAP infection (based on data from Reinders, 1963).

B4.2. Within- and between-herd prevalences in Denmark, Austria, the Netherlands and Sweden

Nielsen and Toft (2009) previously concluded "There are few valid MAP prevalence estimates available in Europe, partly due to problems with test accuracy in target populations, partly due to study designs unsuitable for prevalence inference". This also applied to Austria, where the test accuracies used produced incredible prevalence estimates, the Netherlands were true prevalence estimates were non-interpretable, or different prevalence estimates were produced for the same regions, and in Sweden, where selection bias resulted in lack of credible prevalence estimates (Table C3). Since then, no prevalence studies have been published for those countries. Nielsen (2009) concluded that the between-herd prevalence in Denmark before the onset of the Danish control programme on paratuberculosis in 2006 was approximately 85% based on different studies. By

2013, the between-herd prevalence was estimated to 75% (Verdugo et al., 2015) and the overall cow-level prevalence was estimated to 5.5%, with a median within-herd prevalence in infected herd of 6.8%. These estimates were based on herds within the control programme only.

Table B3. Point estimates and best guesses of prevalences of *Mycobacterium avium* subsp. *paratuberculosis* in Denmark, Austria, the Netherlands and Sweden.

Country	Year	Between-herd	Within-herd	Cow-level	Reference
Denmark	2006	85%			
Denmark	2015	75%	6.5%	5.5%	Verdugo et al., 2015
Austria	1995-97	Best guess: 50-80	Not available	Not available	Nielsen and Toft, 2009
The Netherlands	1999	Best guess: 70%	Not available	Not available	Muskens et al., 2000
Sweden	1995-96	Not available	Not available	Not available	Nielsen and Toft, 2009

The development in the apparent prevalence (antibody prevalence) of the herds enrolled in the voluntary Danish paratuberculosis programme suggests that the within-herd prevalence decreased over the period spanning from 2008 to 2019 (Figure C2). The proportion of herds enrolled also decreased.

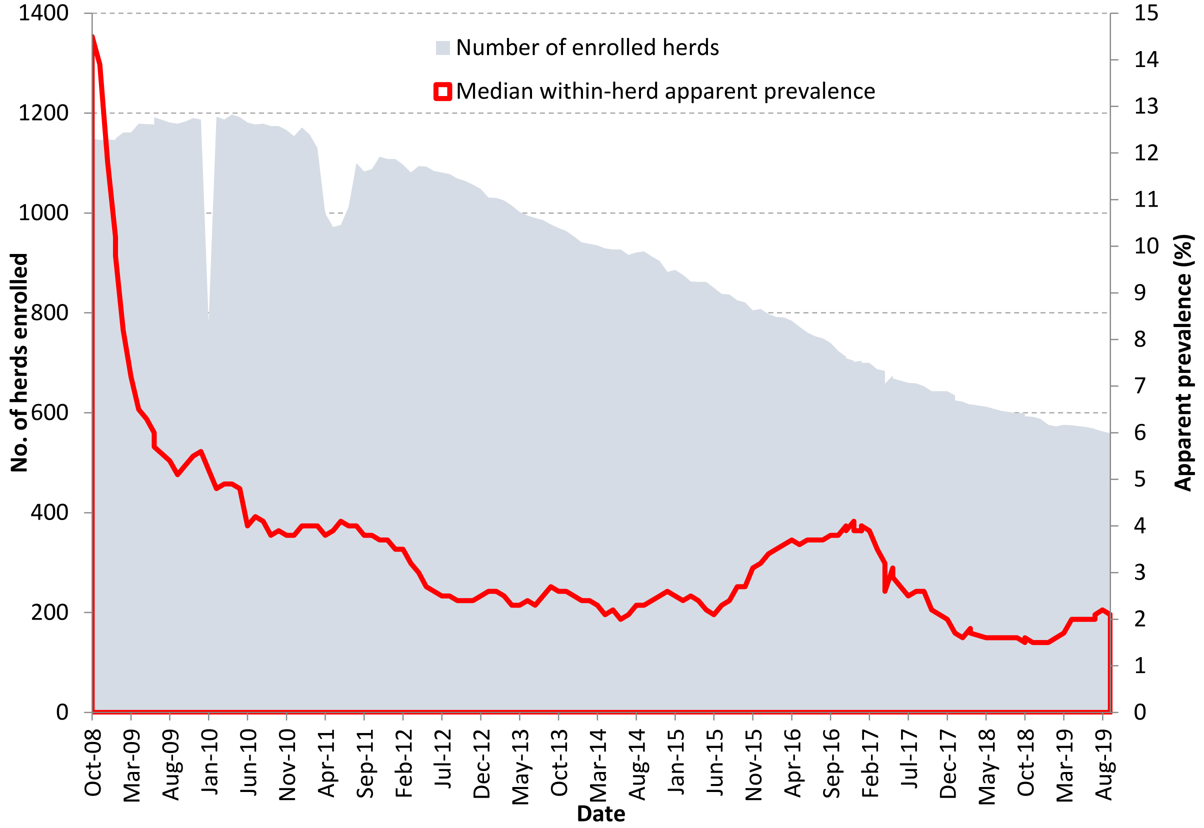

Figure B2. Apparent prevalence (based on ID Screen *Mycobacterium avium* subsp. *paratuberculosis* antibody ELISA) and number of enrolled herds in the Danish control programme on paratuberculosis from October 2008 to October 2019. Data reported weekly in http://kvaegvet.dk/ In October 2008, 4468 dairy herds were recorded, i.e. around 26% of herds. Approximately 29% of dairy herds were enrolled in January 2012 and 20% in August 2019.

B5. References

Anon., 2008. Extract from the records of the court in Odense, Denmark, on 27 June 2008. https://www.landbrugsinfo.dk/Kvaeg/Sundhed-og-dyrevelfaerd/Veterinaert-beredskab/Paratuberkulose/Sider/Domsafsigelse_web.pdf

Brady C, O'Grady D, O'Meara F, Egan J, Bassett H, 2008. Relationships between clinical signs, pathological changes and tissue distribution of Mycobacterium avium subspecies paratuberculosis in 21 cows from herds affected by Johne's disease. Vet Rec. 162:147-52.

Chiodini RJ, Van Kruiningen HJ, Merkal RS, 1984. Ruminant paratuberculosis (Johne's disease): the current status and future prospects. Cornell Vet. 74:218-62.

Dyreværnsloven, 2018. LBK nr 20 af 11/01/2018. Bekendtgørelse af Dyreværnsloven (The Danish Animal Protection Act from 11 January 2018). (https://www.retsinformation.dk/Forms/R0710.aspx?id=197059)

EFSA, 2014. Guidance on Expert Knowledge Elicitation in Food and Feed Safety Risk Assessment. EFSA Journal, 12, 3734.

Espejo LA, Kubat N, Godden SM, Wells SJ, 2013. Effect of delayed exposure of cattle to *Mycobacterium avium* subsp *paratuberculosis* on the development of subclinical and clinical Johne's disease. Am J Vet Res. 74:1304-10.

European Union, 2018. Commission Delegated Regulation (EU) 2018/1629 of 25 July 2018 amending the list of diseases set out in Annex II to Regulation (EU) 2016/429 of the European Parliament and of the Council on transmissible animal diseases and amending and repealing certain acts in the area of animal health ('Animal Health Law'). https://eur-lex.europa.eu/eli/reg_del/2018/1629/oj

Ferrouillet C, Wells SJ, Hartmann WL, Godden SM, Carrier J, 2009. Decrease of Johne's disease prevalence and incidence in six Minnesota, USA, dairy cattle herds on a long-term management program. Prev Vet Med. 88:128-37.

Frössling J, Wahlström H, Agren EC, Cameron A, Lindberg A, Sternberg Lewerin S, 2013. Surveillance system sensitivities and probability of freedom from *Mycobacterium avium* subsp. *paratuberculosis* infection in Swedish cattle. Prev Vet Med. 108:47-62.

González J, Geijo MV, García-Pariente C, Verna A, Corpa JM, Reyes LE, Ferreras MC, Juste RA, García Marín JF, Pérez V, 2005. Histopathological classification of lesions associated with natural paratuberculosis infection in cattle. J Comp Pathol. 133:184-96.

Grant MJ, Booth A, 2009. A typology of reviews: an analysis of 14 review types and associated methodologies. Health Information and Libraries Journal, 26, 91-108. DOI: 10.1111/j.1471-1842.2009.00848.x

Greenhalgh T, Peacock R, 2005. Effectiveness and efficiency of search methods in systematic reviews of complex evidence: audit of primary sources. BMJ. 331:1064-5.

Hagan WA, 1938. Age as a factor in susceptibility to Johne's disease. Cornell Vet. 28: 34-40.

Holstad G, Sigurdardóttir OG, Storset AK, Tharaldsen J, Nyberg O, Schönheit J, Djønne B, 2005. Description of the infection status in a Norwegian cattle herd naturally infected by Mycobacterium avium subsp. paratuberculosis. Acta Vet Scand. 46:45-56.

Jubb T, Galvin J, 2000. Herd testing to control bovine Johne's disease. Vet Microbiol. 77:423-8.

Jubb TF, Galvin JW, 2004a. Effect of a test and control program for Johne's disease in Victorian beef herds 1992-2002. Aust Vet J. 82:164-6.

Jubb TF, Galvin JW, 2004b. Effect of a test and control program for bovine Johne's disease in Victorian dairy herds 1992 - 2002. Aust Vet J. 82:228-32.

Khol JL, Baumgartner W, 2014. 8 years of compulsory measures against clinical paratuberculosis in Austria - a success? Proceedings of the 4th ParaTB Forum. Bulletin of the IDF No. 475/2014 p. 5-9.

Khol JL, Pinedo PJ, Buergelt CD, Neumann LM, Baumgartner W, Rae DO, 2012. The collection of lymphatic fluid from the bovine udder and its use for the detection of *Mycobacterium avium* subsp. *paratuberculosis* in the cow. J Vet Diagn Invest. 24:23-31.

Kudahl A, Nielsen SS, Sørensen JT, 2004. Relationship between antibodies against *Mycobacterium avium* subsp. *paratuberculosis* in milk and shape of lactation curves. Prev Vet Med. 62:119-34.

Kudahl AB, Nielsen SS, 2009. Effect of paratuberculosis on slaughter weight and slaughter value of dairy cows. J Dairy Sci. 92:4340-6.

Larsen AB, Merkal RS, 1968. The effect of management on the incidence of clinical Johne's disease. J Am Vet Med Assoc. 152:1771-3.

Matthews HT, 1947. On Johne's disease. Vet Rec. 59:397-401.

More S, Bøtner A, Butterworth A, Calistri P, Depner K, Edwards S, Garin-Bastuji B, Good M, Gortázar Schmidt C, Michel V, Miranda MA, Nielsen SS, Raj M, Sihvonen L, Spoolder H, Stegeman JA, Thulke H-H, Velarde A, Willeberg P, Winckler C, Baldinelli F, Broglia A, Zancanaro G, Beltrán-Beck B, Kohnle L, Morgado J, Bicout D, 2017. Scientific Opinion on the assessment of listing and categorisation of animal diseases within the framework of the Animal Health Law (Regulation (EU) No 2016/429): paratuberculosis. EFSA J. 15:4960.

Mortier RA, Barkema HW, Orsel K, Muench GP, Bystrom JM, Illanes O, De Buck J, 2015. Longitudinal evaluation of diagnostics in experimentally infected young calves during subclinical and clinical paratuberculosis. Can Vet J. 56:1266-70.

Muskens J, Barkema HW, Russchen E, van Maanen K, Schukken YH, Bakker B, 2000. Prevalence and regional distribution of paratuberculosis in dairy herds in The Netherlands. Vet. Microbiol. 77, 253–261.

Nielsen SS, Toft N, 2009. A review of prevalences of paratuberculosis in farmed animals in Europe. Prev Vet Med. 88:1-14.

Nielsen SS, Toft N, 2011. Effect of management practices on paratuberculosis prevalence in Danish dairy herds. J Dairy Sci. 94:1849-57.

Paolicchi F, Perea J, Cseh S, Morsella C, 2013. Relationship between paratuberculosis and the microelements copper, zinc, iron, selenium and molybdenum in beef cattle. Braz J Microbiol. 44:153-60.

ParaTB Forum, 2016. Nantes, France. Proceedings of the 5th, Paratuberculosis Forum. Bulletin of the IDF No. 484/2016.

ParaTB Forum, 2014. Parma, Italy. Proceedings of the 4th ParaTB Forum. Bulletin of the IDF No. 475/2014.

ParaTB Forum, 2012. Sydney, Australia, February 4, 2012. Proceedings of the 3rd ParaTB Forum. Bulletin of the IDF No. 460/2012.

ParaTB Forum, 2009. Minneapolis, Minnesota, USA. August 8, 2009.Proceedings of 2nd paratuberculosis Forum. Bulletinf of the IDF No. 441/2009.

ParaTB Forum, 2007. Shanghai, China, 2007. Proceedings of the 1st ParaTB Forum. Bulletin of the IDF No. 410/2007

Patterson DS, Allen WM, Berrett S, Ivins LN, Sweasey D, 1968. Some biochemical aspects of clinical Johne's disease in cattle. Res Vet Sci. 9:117-29.

Patterson DS, Allen WM, Berrett S, 1965. Plasma enzymes in clinical Johne's disease. Vet Rec. 77:1287-9.

Prewett LP, 1950. Johne's disease; epidemiology and control under Victorian conditions. Aust Vet J. 26:213-6.

Raizman EA, Wells SJ, Godden SM, Fetrow J, Oakes JM, 2007. The associations between culling due to clinical Johne's disease or the detection of *Mycobacterium avium* subsp. *paratuberculosis* fecal shedding and the diagnosis of clinical or subclinical diseases in two dairy herds in Minnesota, USA. Prev Vet Med. 80:166-78.

Reinders JS, 1963. Bestrijding van klinische paratuberculose bij runderen (Control of clinical Johne’s disease in cattle). Thesis, University of Utrecht, the Netherlands, 194 pp.

Shahmoradi AH, Arefpajohi R, Tadayon K, Mosavari N, 2008. Paratuberculosis in Holstein-Friesian cattle farms in Central Iran. Trop Anim Health Prod. 40:169-73.

Van Amstel SR, 1984. Observations on the symptomatology and diagnosis of clinical cases of Johne's disease. J S Afr Vet Assoc. 55:45-6.

Verdugo C, Toft N, Nielsen SS, 2015. Within- and between-herd prevalence variation of *Mycobacterium avium* subsp. *paratuberculosis* infection among control programme herds in Denmark (2011-2013). Prev Vet Med. 121:282-7.

Weber MF, Aalberts M, Schukken YH, 2016. An update on milk quality assurance for paratuberculosis in Dutch dairy herds. Bulletin of the International Dairy Federation 484, 23-30.

Whitlock RH, Buergelt C, 1996. Preclinical and clinical manifestations of paratuberculosis (including pathology). Vet Clin North Am Food Anim Pract. 12:345-56.

Ågren E, Frössling J, Holmström A, Larsson B, 2012. Demonstrating freedom from MAP infection in Swedish cattle, what´s next? Proc. 3rd ParaTB Forum 4 February 2012, p. 76-78.

C. Aujeszky´s disease in swine - review of clinical manifestations.

By Nina Dam Otten

C1. Summary

Aujeszky´s disease in swine - review of clinical manifestations.

Aujeszky's disease or pseudorabies is caused by porcine herpes virus 1, which gives rise to a highly contagious clinical condition with high morbidity for all age groups, while mortality is decreasing with age and dependent on the virulence of the virus in question. However, piglet mortality is most often at 100 %. Infection with Aujeszky's primarily results in neurological clinical manifestations with e.g. weakness, fever and convulsions leading to death of the piglets. Weaned pigs are most often affected by respiratory symptoms with an increased risk of secondary bacterial infections. In sows, reproductive disorders such as abortion or mummification of fetuses, weak-born pigs and agalactiae are also seen, which, together with intrauterine infection of the fetuses, reduces the survival chances of newborn pigs. This review describes the individual clinical syndromes for each age group and evaluates the welfare consequences of the severity and duration of the syndromes.

C2. Introduction of the disease

Aujeszky´s disease (AD)/pseudorabies or 'Mad itch' is a latent infection with suid Herpesvirus 1 in swine acting as reservoir hosts for spontaneous transmission to other species, e.g. cattle and pets regarded as dead end hosts. Both morbidity and mortality are age dependent. Piglets suffer welfare impacts due to the severe CNS impairment of encephalitis leading to painful symptoms like convulsions and opisthotonus with fatal outcomes. Weaned and adult pigs are more prone to production losses as they rather suffer from respiratory and reproductive impairment e.g. abortion, agalactia and smaller litter sizes than the occasional fatal CNS involvement leading to sudden death. Secondary bacterial infections further enhance the negative welfare consequences and production losses. Swine do not show signs of local pruritus leading to the 'mad itch' described in ruminants.

Effective vaccines reducing viral shedding and improving survival of especially piglets are available. However, the use of these vaccines is not allowed in countries that have been declared free form disease.

C3. Methods

C3.1. Clinical signs, severity, duration, and mortality/case-fatality

A systematic review within PubMed was performed using the search terms:

(Aujeszky´s disease or pseudorabies and (sow* or pig* or swine) and (clinical sign* or clinical disease or death or mortality)) 383 hits -> + not (vacc*) 201

Exclusion criteria for the systematic review: a) not original study of clinical disease (this had to be evident from abstract); b) not cattle; c) language: non-English; d) comorbidities; and not vaccine/vaccination.

A total of 383/201 hits were identified at the search on August 26-29 2019. After assessment of the titles/abstracts and inclusion of publications identified by snowballing, a total of 15 peer-reviewed publications together with a number of official reports were included.

C3.2. Within- and between-herd prevalences /incidences in Denmark, Germany, Sweden, The Netherlands UK and Spain

Based on the before mentioned literature search all available information on within- and between herd prevalences of AD were extracted for the relevant countries. The EFSA report on Aujeszky´s disease (More et al., 2017) also provided relevant information on several issues, amongst these prevalence estimates. Furthermore, links to official and/or federal websites regarding the respective countries eradication programme were also investigated for possible references on both historical and present prevalence estimates.

C4. Results

C4.1. Clinical signs, severity, duration, and mortality/case-fatality

Consequences of natural occurring pseudorabies infections vary across the virulence of strains, age groups of infected animals and previous exposure (Mettenleiter et al., 2019; Lee and Wilson, 1979; Kluge et al., 1999). Hence, the following section will describe the typical infection characteristics mentioned above within a given age group. Whereas strain virulence determines the type of infection; characterized by highly virulent strains inducing neurological signs and less virulent strains affecting the respiratory tract (Mettenleiter et al., 2019); the route of infection and the age and immune status of the animal determines the severity and the outcome of the infection (Nauwynck, 1997). Incubation periods have been reported lasting between 1-11 days. Since viral shedding occurs in the nasal discharge from infected animals from the onset of clinical signs and lasts up to 21 days (Radostits et al., 2007; Kluge et al., 1999; Nauwynck, 1997), transmission occurs rapidly and morbidity often reaches 100 % and acute outbreaks may last for 1-2 months.

C4.1.1. Piglets

Suckling piglets are highly susceptible to AD infections with a short incubation period of only 2 to 4 days (Pomeranz et al., 2005; Kluge et al., 1999; Inch, 1998). The initial febrile response leads to listlessness and reluctance to nurse and symptoms rapidly progress into more specific CNS signs like trembling, hypersalivation, incoordination of the hindlimbs leading lateral recumbency and further progressing into convulsions and opisthotonus. Due to the extreme challenges caused by the viral encephalitis; piglets succumb within 24 to 36 hours after the onset of CNS signs. Mortality is as high as a 100 % in piglets up to two weeks of age (Lee & Wilson, 1979; Pomeranz et al., 2005; Inch, 1998).

C4.1.2. Weaners and finishing pigs

As the pathogenicity declines with age, signs of the infection in weaned pigs (> 3-4 weeks of age) are less severe. Animals may still show CNS signs such as trembling, tremors or incoordination of the hindlimbs; however, mortality of 50 % are seen and these decrease further as pigs develop immunity with increasing age. In pigs aged 4-6 month mortality rates typically are 5 % (Radostits et al., 2007; Nauwynck, 1997) and symptoms of infection rather present as febrile responses, depression/anorexia and/or vomitus. Respiratory signs ranging from nasal discharge to severe dyspnea may also become evident, as pigs no longer necessarily die from the neurological impact of the AD infection. Nonetheless, due to the higher stocking density among weaners and finishers the risk of contracting secondary bacterial infections increases.

C4.1.3. Adult pigs

Neurological signs may be present in adult pigs; however, the hallmarks of the infection amongst animals within this age group are respiratory signs. Initially, the clinical manifestations of AD are rhinitis with sneezing and nasal discharge later progressing into pneumonia (Kluge et al., 1999). In up to 20 % of the cases reproductive failure with varying outcome is seen. Transplacental virus transmission within the first trimester causes necrotizing placentitis and endometritis leading to abortion and/or mummification or reabsorption of fetuses (Pomeranz et al., 2005). Infections in the second and third trimester also lead to stillbirth or to weak piglets dying shortly after birth. Piglet death may be due to necrotic lesions within vital organs (Kluge & Maré, 1976) or to malnutrition caused by the affected sow having agalactiae. Affected sows will become infertile. In boars scrotal edema may be exhibited (Mettenleiter et al., 2019).

Table C1. Clinical manifestations/disease stages amongst different age groups of Aujeszky's disease infections, duration and implications for animal welfare

Clinical manifestation	Typical signs	Frequency of signs. Morbidity /mortality	Duration	Welfare implications
Piglets in utero	ENCEPHALITIS<br>Shaking/shivering<br>Sudden death	Mort: 100%	Death within two days post partum	Severe
Piglets (< 3 weeks)	FEBRILE RESPONSE<br><br>ENCEPHALITIS<br>lethargy, weakness/appetite loss,	High morbidity and mortality rates: 100%	2-3 days<br>Death can occur within 12 hours from onset of clinical signs	Severe<br><br>Encephalitis symptoms are very painful

	incoordination, convulsions, (vomitus, diarrhea)		
Weaners & finisher pigs (> 3 weeks)	FEBRILE RESPONSE<br><br>ENCEPHALTIS<br>Loss of appetite<br>Somnolence<br>Trembling/convulsions<br>paralysis<br><br>RESPIRATORY SIGNS<br>Sneezing, nasal discharge, coughing, dyspnea	Mort: 10-20 %<br>5 % (Radostits et al., 2007)<br><br>Mort: 50 % (Nauwynck, 1997)	Severe<br><br>Severe infections cause welfare problems due to painful consequence of encephalitis and subsequent mortality<br><br>Possible sequelae: pressure sores (Segalés et al., 2003)
Adults	FEBRILE RESPONSE<br><br>RESPIRATORY<br><br>ENCEPHALITIS (occasionally)<br>Incoordination of hindlimbs<br><br>ABORTION<br>Vaginal discharge/mummification<br>Agalactia<br>Weak piglets	Mort: less than 5 %<br>(depending on virulence of strain)<br><br>1-2 % (Pomeranz et al.,2005; Mettenleiter et al., 2019)	Moderate<br><br>Possible sequelae:<br><br>Weight loss Secondary bacterial infections<br>Weak piglets<br>Pressure sores

C4.2. Within- and between-herd prevalences /incidences in Denmark

Clinical AD infections were described in Danish pigs for the first time in 1964. While the disease was eradicated from breeding herds by 1969, the remaining herds still struggled with infections until a joint eradication campaign by the industry and the authorities was launched in 1980. By 1986, AD was eradicated from Denmark apart from epidemic incursions along the Danish-German border (Christensen et al., 1993). The eradication program was successful and by 1991 Denmark obtained the official disease-free status.

In 2017 the Danish surveillance scheme found 1 test-positive among 32,832 samples tested for antibodies to AD by ELISA. This particular animal turned out to be an imported animal. Further six animals tested positive at export control. However, all seven suspicions were revoked by the subsequent neutralization test (DTU Veterinærinstituttet, 2018).

C4.3. Within- and between-herd prevalences in selected other countries

At present Denmark's closest neighboring countries Germany and Sweden share the AD disease free status. Initially, the AD prevalence in Germany was as high as 70 % seropositive sows in 1993, but due the implementation of a vaccination and eradication program this was reduced to 1 % over a five year period. Germany obtained disease free status in 2002. Since wild boars act as a natural infection reservoir for AD, disease monitoring within the wild boar population is performed on federal level. Reported prevalence estimates range between 2-29% and together with hunting dogs being regularly infected, AD is still a threat to pig populations on both sides of the terrestrial border between Denmark and Germany.

The Swedish pig population showed a general lower between-herd prevalence of 9 % in 1990 (Andersson et al., 1997), when the eradication program was launched and, therefore, quickly reached the disease free status in 1996.

AD had been endemic in the Netherlands when the eradication program was launched in 1993. Seroprevalences in sows between 10-32 % and 1-18 % in finishing pigs in 1994 declined to 0.5 % for both age groups by 1998 (Elbers et al., 2000). Disease free status was not obtained until 2009.

In the UK the AD eradication program was initiated in 1983 where the incidence of AD peaked with 0.18 % between pig herds (Onneile et al., 2011). Great Britain has officially been disease free since 1991, while Northern Ireland obtained this status in 2012 (DEFRA, 2019).

Sporadic cases of infected domesticated herds still occur frequently in the southern European regions like Spain and France. Especially in regions, where contact to the wild boar population is present, these cases still occur. The seroprevalence among the wild boar population in Spain was at 30% in 2017 (MAGRAMA, 2018). Lately, in April 2019 five animals from two different domestic pig herds in Southern France were confirmed as clinical cases, due to contact with wild boars (DEFRA, 2019 a). Müller et al. (2010) reported AD virus being isolated from wild boars and hunting dogs in Germany, Austria, France, Spain, Italy, Slovakia and Hungary.

Table C2. Summary table of seroprevalences of infections with Aujeszkys´disease in endemic areas prior to the initiation of control programs

Infection stage	Population level	
	Individual level (in infected herds)	Herd level (presence of any animal with infection stage)
Sows	1-18%	0.2-9%
Weaners & finishers	10-70 %	

C5. References

Andersson H., Lexmon Å., Robertsson JÅ., Lundeheim N., Wierup M. (1997). Agricultural policy and social returns to eradication programs: the case of Aujeszky's disease in Sweden. *Preventive Veterinary Medicine* 29(4):311-328

Baskerville A. (1972). The influence of dose of virus on the clinical signs in experimental Aujeszky’s disease in pigs, *British Veterinary Journal,* 128:394-401

Boklund A., Dahl J., Alban L. (2013). Assessment of confidence in freedom from Aujeszky’s disease and classical swine fever in Danish pigs based on serological sampling—Effect of reducing the number of samples. *Preventive Veterinary Medicine* 110: 214–222

Bosman KJ., Mourits MCM., Oude Lansink AGJM., Saatkamp HW. (2013). Minimization of the Impact of Aujeszky’s Disease Outbreaks in The Netherlands: A Conceptual Framework. *Transboundary and Emerging Diseases,* 60(4):303-314

Christensen LS., Mousing J., Mortensen S., Soerensen KJ., Strandbygaard SB., Henriksen CA. & Andersen JB. (1990). Evidence of long distance airborne transmission of Aujeszky's disease (pseudorabies) virus. *Veterinary Record*, 127, 471–474

Christensen, L.S., Mortensen, S., Bøtner, A., Strandbygård, B.S., Rønsholt, L., Henriksen, C.A. & Andersen, J.B. (1993). Further evidence of long distance airborne transmission of Aujeszky's disease (pseudorabies) virus. *Veterinary Record* 27: 317-321

DEFRA (2019 a). “Aujeszky’s disease in domestic swine in France “, Disease Outbreak Report, available online: https://assets.publishing.service.gov.uk/government/uploads/system/uploads/attachment_data/file/799190/poa-auj-france.pdf

DEFRA (2019 b). “Aujeszky’s disease in wild boar in Finland “, Disease Outbreak Report, available online:

https://assets.publishing.service.gov.uk/government/uploads/system/uploads/attachment_data/file/840218/poa-auj-finland.pdf

Elbers , ARW., Braamskamp , J., Dekkers , LJM, Voets , R., Duinhof , T.,Hunneman, WA. & JA. Stegeman. (2000). Aujeszky's disease virus eradication campaign successfully heading for last stage in the Netherlands. *Veterinary Quarterly*, 22:2, 103-107, DOI: 10.1080/01652176.2000.9695034

Gustafson DP. (1986) Pseudorabies.In: Diseases of Swine (AD Leman et al, eds), Ames, Iowa State University Press, 274-289

Kluge, JP., Beran ,GW., Hill, HT. & KB. Platt. (1999). Pseudorabies (Aujeszky's disease), p. 233-46. *In* B. E. Straw, S. D'Allaire, W. L. Mengeling, and T. J. Taylor (ed.), Diseases of swine, 8th ed. Iowa State University Press, Ames, Iowa.

Leontides L., Ewald C., Mortensen S., Willeberg P. (1995). Factors associated with the seroprevalence of Aujeszky's disease virus in seropositive breeding herds of Northern Germany during area-wide compulsory vaccination. *Preventive Veterinary Medicine* 23:73-85

MAGRAMA, Ministry of Agriculture, Food and Environment (2017): "Resultados del programma de vigilancia de PPA, PPC, EVP y EA en jabalíes para el año 2016". Available at: https://www.mapa.gob.es/es/ganaderia/temas/sanidad-animal-higiene-ganadera/resultadosdelultimoprogramanacionaldevigilanciadejabalies_tcm30-376479.pdf

MAGRAMA, Ministry of Agriculture, Food and Environment (2018): "Enfermedad de Aujezsky" Availbale at: https://www.mapa.gob.es/es/ganaderia/temas/sanidad-animal-higiene-ganadera/sanidad-animal/enfermedades/aujezsky/enf_aujezsky.aspx

Mettenleiter TC., Ehlers B., Müller T., Yoon K-J. & Teifke J.P. 2019. Suid alphaherpesvirus 1 (Aujeszky's disease virus; pseudorabies virus), p. 551-561. *In* Zimmermann J. (ed.), Diseases of swine, 11th ed. John Wiley Sons, Inc., Hoboken, NJ.

More S, Bøtner A, Butterworth A, Calistri P, Depner K, Edwards S, Garin-Bastuji B, Good M, Gortázar Schmidt C, Michel V, Miranda MA, Nielsen SS, Raj M, Sihvonen L, Spoolder H, Stegeman JA, Thulke H-H, Velarde A, Willeberg P, Winckler C, Baldinelli F, Broglia A, Zancanaro G, Beltrán-Beck B, Kohnle L, Morgado J, Bicout D, 2017. Scientific Opinion on the assessment of listing and categorisation of animal diseases within the framework of the Animal Health Law (Regulation (EU) No 2016/429): paratuberculosis. EFSA J. 15:4960.

Muller T., Klupp BG., Freuling C., Hoffmann B., Mojcicz M., Capua I., Palfi V., Toma B., Lutz W., Ruiz-Fon F., Gortázar C., Hlinak A., Schaarschmidt U., Zimmer K., Conraths FJ., Hahn EC. & Mettenleiter TC. (2010). Characterization of pseudorabies virus of wild boar origin from Europe. *Epidemiology and Infections*, 138, 1590–1600

Nauwynck, HJ. (1997). Functional aspects of Aujeszky's disease (pseudorabies) viral proteins with relation to invasion, virulence and immunogenicity. *Veterinary Microbiology* 55(1-4):3-11

Onneile, O.P., Peiso O.O., Bronsvoort B.M.dC., Handel I.G., Volkova V.V. (2011). A Review of Exotic Animal Disease in Great Britain and in Scotland Specifically between 1938 and 2007. *PLoS ONE* 6(7): e22066. https://doi.org/10.1371/journal.pone.0022066

Radostits, O., Gay, C., Hinchcliff, K., and Constable, P. (2007). Pseudorabies (Aujeszky´s Disease), p1394-1404. *In* Veterinary Medicine, 10th ed., Saunders Ltd

Segalés J., Chianini F., Cortés R., and Domingo M. (2003). Neurological disorders associated with Aujeszky's disease virus infection in fattening pigs. *Veterinary Record* 153(8):240-241.

Stegeman A. (1997): "Aujeszky ' s disease (pseudorabies) virus eradication campaign in the Nether-lands". *Veterinary Microbiology* 55:175- 180

Vicente-Rubiano M., Martinez-Lopez B., Sanchez-Vizcaino F., Sanchez-Vizcaino JM. (2014): "A new approach for rapidly assessing the risk of Aujeszky`s disease reintroduction into a disease-free spanish territory by analysing the movement of live pigs and potential contacts with wild boar". *Transboundary and Emerging Diseases,* 61(4):350-61

DTU Veterinærinstituttet (2018): "Årsrapport 2017 Laboratorieundersøgelser af materiale fra SVIN på DTU Veterinærinstituttet og SEGES Laboratoriet i Kjellerup" available online

Wittmann G. (1986): "Aujeszky's disease". *Revue scientifique et technique (International Office of Epizootics),* 5(4):959-977

Wittmann G. (1991): "Spread and control of Aujeszky's disease (AD)". *Comparative Immunonology, Microbiology and Infectious Diseases,* 14:165-173

Veijalainen, PM-L. & Tapiovaara, H. (2000). No Aujeszky's disease or PRRS in Finland. *Veterinary Research,* 31(1): 153-154

D. Porcine Reproductive and Respiratory Syndrome in Pigs – systematic search and review of clinical manifestations

By Anne Kirstine Manly Andersen and Matt Denwood

D1. Summary

Porcine Reproductive and Respiratory Syndrome in Pigs – systematic search and review of clinical manifestations.

Porcine Reproductive and Respiratory Syndrome (PRRS) is an extremely widespread viral pathogen of pigs that is associated with substantial economic costs worldwide. In affected countries with no control measures, which represents the majority of European states including the UK, Netherlands and Spain, up to 90% of animals are affected in some way by the disease. Although clinical symptoms in adult animals are most frequently mild and transient, the disease is associated with substantial welfare concerns in piglets. Given the high between-herd prevalence and extremely high morbidity associated with PRRS, the potential overall welfare implications of the disease are therefore substantial.

D2. Introduction

Porcine Reproductive and Respiratory Syndrome (PRRS) is a relatively recently described syndrome, having first been reported in the United States and Canada in 1987, and known variously as Mystery Swine Disease, Swine Infertility and Respiratory Syndrome, Blue-ear Pig Disease, and Porcine Epidemic Abortion and Respiratory Syndrome (S. A. Hopper et al. 1992, Wensvoort 1993, Done et al. 1996). Following identification in Germany and the Netherlands in 1990, PRRS spread rapidly through Europe as well as the Far East (Done et al. 1996, Wensvoort 1993), and has become one of the most economically important infectious diseases in pig production (Pejsak & Markowska-Daniel 1997, Nathues et al. 2017).

PRRS is caused by the Lelystad virus or PRRS virus; an RNA virus with a high rate of mutation leading to several distinct circulating strains of the virus (Rossow 1998), of which the first was isolated in the Netherlands in 1991 (Wensvoort 1993). Two strains are known to be circulating in Denmark: the European type PRRS-1 and the American type PRRS-2 (Antunes et al. 2015). Of these, the American type is associated with somewhat higher pathogenicity (Jeong et al. 2018), although less than that when it was originally identified in the United States (Blaha et al. 2000). PRRS virus can be transmitted horizontally through contaminated body fluids, especially through direct contact during social behaviour, as well as vertically by infecting foetuses in utero (More et al. 2017). The most frequently observed clinical symptoms of PRRS can be ascribed to two main syndromes: respiratory and reproductive. The respiratory syndrome is most often observed during the initial stages of infection within a herd, and includes a wide variety of symptoms typically including fever and dyspnoea, but also potentially coughing, sneezing, and sudden death. It is most prominent in nursery piglets, but can be seen in all age groups (Rossow 1998, Hopper et al. 1992, Baron et

al.1992, Done & Paton 1995, Pejsak & Markowska-Daniel 1997). The reproductive syndrome is associated with premature farrowing, abortions, increased numbers of stillbirths, mummifications, weak born piglets, and an increase in reported returns to service (Jeong et al. 2018).

Once the initial epidemic phase of infection has subsided, the endemic state of PRRS within a herd is often subclinical, meaning that the respiratory syndrome is no longer observed and the herd productivity returns to acceptable levels within a few months (Nodelijk 2002). However, an important characteristic of PRRS is the variation in the range of clinical effects seen both at the herd and individual animal level (Wensvoort 1993, Done et al. 1996), and reproductive problems are recurrent in endemically infected herds (Nodelijk et al. 2003). It is also important to note that unless regional efforts are made to control an epidemic outbreak of PRRS, the infection typically becomes endemic within the population and is subsequently associated with an increased risk of infection and subsequent clinical disease for other herds within that area (Antunes et al. 2015).

PRRS has received increasing interest in recent years, and various control efforts have been implemented in an effort to limit the spread of infection. However, the motivation for control of the disease appears to be primarily driven by the economic consequences of infection, in particular the reproductive syndrome. The overall aim of this work was therefore to assess the potential welfare implications of PRRS infection within Denmark, with specific objectives to (a) summarise the clinical signs associated with different syndromes of PRRS infection, (b) estimate the within-herd prevalence of these syndromes, and (c) estimate the between-herd prevalence of infection within a number of EU countries with different approaches to control of the infectious agent.

D3. Methods

D3.1. Clinical signs, severity, duration, and mortality/case-fatality

A systematic review within PubMed was performed on 23rd September 2019 using the search terms:

(“Blue Ear” OR PRRS OR “porcine reproductive and respiratory syndrome”) AND ("clinical sign*" OR “clinical disease OR death OR mortality) AND (pig* OR swine OR piglet* OR weaner*)

Abstracts from these were assessed by one reviewer and the following exclusion criteria were used: non-English or non-German language, not on clinical disease or signs, paper not from Europe, Canada, or the United States of America, experimental, vaccination or genetic studies. Review articles were included. All information regarding description, symptoms and severity of clinical signs was extracted, summarized and tabulated.

D3.2. Within- and between-herd prevalence/incidence in Denmark, Sweden, the United Kingdom, the Netherlands and Spain

Recent information on the within- and between-herd prevalence of PRRS within various EU countries was identified from the literature search as summarised above, with a particular emphasis on estimates from Denmark, Sweden, United Kingdom, the Netherlands and Spain. Information was

also extracted directly from a highly relevant European Food Safety Authority (EFSA) report (More et al. 2017).

D4. Results

D4.1. Clinical signs, severity, duration, and mortality/case-fatality

The search produced a total of 638 hits, of which 34 articles were deemed to match the inclusion criteria. These articles focused to a large extent on the economic consequences of PRRS, and within-herd dynamics of infection as relevant to control of the disease. Although not the primary interest of this report, a brief summary of these findings is given here. Initial infection with PRRS virus is described as being associated with a period of respiratory symptoms followed by a period of reproductive disorders (Baron et al. 1992). A small study in 1994 showed that sentinel pigs introduced to a herd already infected with PRRS developed fever, and that pigs in the nursery section were affected for a longer time than were finishers (Bilodeau et al. 1994). The virus can persist in the individual animal for up to six months post infection, and in-utero infection has been demonstrated for piglets from a sow that has been sero-negative for several months (Wensvoort 1993). Experimental studies, however, suggest that persistent infection rarely last longer than 200 days (Lunney et al. 2010). Infection is therefore more likely to die out in small herds relative to bigger herds, where the virus can circulate and therefore persist (Wensvoort 1993, Nodelijk et al. 2003). Maintaining a closed herd and unidirectional flow of pigs and humans within the farm seems to be efficient in stabilizing an infected herd (i.e. weaning PRRS-negative pigs from seropositive sows) in combination with vaccination (Berton et al. 2017).

In general, the clinical signs were described in less detail than were the issues summarized above, but sufficient detail was given to these aspects in order to be able to group the clinical signs into syndromes associated with PRRS infection. These syndromes are given below and summarized in Table D1. However, it is important to note that the severity and duration, as well as the clinical signs themselves, vary greatly between herds. In particular, some infections lead to severe disease and others remain subclinical (Baron et al. 1992, Goyal 1993, Reeth 1997, Mengeling et al. 2000, Young et al. 2010).

D4.1.1. Epidemic syndrome of sows and boars

In the acute stages of infection in sows there are consistent reports of anorexia, fever, lethargy and respiratory difficulties, although the severity of these signs varies between affected sows. Anorexia develops in late-stage gestation sows before those in earlier stages of gestation, but as the infection spreads through the herd anorexia can be seen in all sows, including to a limited extent in dry sows. When a large herd is infected a ”rolling inappetence” can often be observed as the infection progresses through the herd (Goyal 1993, Nodelijk 2002). Fever followed by lethargy and respiratory signs such as coughing, sneezing, and hyperpnoea are also seen in the acute stages of infection of a naïve herd. In about 1% of cases a cyanotic blue or reddish discolouration of the ears, abdomen and vulva can be observed for a short period (Baron et al. 1992, Hopper et al. 1992,

Goyal 1993, Done & Paton 1995, Pejsak & Markowska-Daniel 1997, Sinn et al. 2016, Balasuriya & Carossino 2017).

In boars, infection with PRRS virus most often goes unnoticed. Rarely, animals show transient clinical signs similar to the sows such as fever, lethargy, and anorexia and in addition sometimes a loss of libido (Done et al. 1996). However, virus can still be transmitted through semen, thus making subclinical infected boars a great risk of further spreading of the virus, if the semen is used for artificial insemination (Prieto & Castro 2005, Nathues et al. 2016). This has been demonstrated by an outbreak in Switzerland in 2012 and in a more recent case of PRRS infection involving a Danish boar stud (Prieto & Castro 2004, SEGES 2019).

D4.1.2. Endemic syndrome of sows

After the acute phase of infection has passed, infection is primarily manifest as reproductive problems such as an increase in abortions, premature births, stillbirths, mummifications, and returns to service (Jeong et al. 2018). No other clinical signs are consistently reported.

D4.1.3. Nursery syndrome

Clinical signs associated with infection of piglets in utero or shortly after birth include poor growth rate, anorexia, fever, respiratory distress, diarrhoea, anaemia, roughened hair coats, periorbital oedema, conjunctivitis, domed head shape, and muscle tremors. Additional signs including statue-like standing, splay-leggedness or posterior paresis that will eventually turn into weakness and ataxia have also been reported (Baron et al. 1992, Hopper et al. 1992, Wensvoort 1993, Done & Paton 1995, Pejsak & Markowska-Daniel 1997, Sinn et al. 2016,). Severe haemorrhage or bruising can be seen at navel sites and after injection or tail docking (Hopper et al. 1992). Nursery problems are often preceded by an acute infection of PRRS, but may also be cyclical with alternating problem periods and periods of improvement (Done & Paton 1995). Another important aspect is immunomodulation associated with PRRS infection, which can predispose the animal to secondary infections (Lewis et al. 2007, Sinn et al. 2016, Dee et al. 2018).

D4.1.4. Weaner/finisher syndrome

The most common clinical signs are short periods of anorexia, respiratory disorders and transient discolouration of the ears (Hopper et al. 1992, Wensvoort 1993), although in general these symptoms are relatively mild.

Table D1. Overview of the clinical syndromes associated with PRRS, including typical duration, morbidity and mortality rates within infected herds.

Syndrome	Typical signs	Frequency of signs	Duration	Welfare Implications
Epidemic *(sows and*	Anorexia, fever, lethargy, respiratory difficulties, cya-	*Morbidity:* 5-50 % (Done et al. 1996)	2 days – 2 weeks (Goyal 1993)	Moderate

boars)	nosis	*Mortality:* 0-1.5 % (Hopper et al. 1992)		
Endemic *(sows)*	Reproductive problems including abortion, stillbirth, and returns to service	*Morbidity:* ~100% *Mortality:* Negligible (Jeong et al. 2018)	Abortions 10 weeks – 6 months (Rossow 1998) Performance may return to normal or stay slightly below pre-infection levels (Goyal 1993)	Mild
Nursery	Poor growth, anorexia, fever, respiratory distress, diarrhoea, anaemia, congenital abnormalities, weakness, ataxia, haemorrhage, immunomodulation	*Morbidity:* Up to 80% *Mortality:* Highly variable, but potentially up to 100% of the clinically affected animals (Done and Paton 1995)	Highly variable depending on clinical signs from a few days to the (potentially reduced) lifetime of the piglet (Blaha et al. 2000, Nodelijk 2002)	Severe
Weaner and finisher	Transient anorexia, respiratory disorders, and discolouration of the ears	*Morbidity:* 4-47 % (de Paz et al. 2015) *Mortality:* Negligible (Hopper et al. 1992)	5-7 days (Goyal 1993)	Mild

D4.2. Within- and between-herd prevalence/incidence in Denmark, Sweden, the United Kingdom, the Netherlands and Spain

PRRS is currently enrolled in the Danish SPF programme, so relatively good data is available in terms of between-herd prevalence within Denmark. Ongoing monitoring suggests that herd-level PRRS positivity has been reduced from 44% of Danish SPF herds in 2007 to 26% of Danish SPF herds in 2018 (SEGES 2019). Studies of all Danish swine herds indicate a seropositive rate of approximately 40% in 2015 and 35% in 2017 (Antunes et al. 2015, Kvisgaard et al. 2017). It is therefore reasonable to assume that PRRS virus is currently present on approximately 1/3 of Danish pig herds, or at a somewhat lower rate on SPF herds. This can be contrasted with higher herd-level

prevalence estimates for other EU countries with no comparable control programme, for example 80-95% in Italy and Spain.

The available data on within-herd prevalence of infection is somewhat harder to interpret. For example, an outbreak of PRRS in France 1997 reported an extremely large range of within-herd prevalence of 0% - 95% (Le Potier et al. 1997). Furthermore, data from a range of European contexts reveals large differences in case-morbidity rates depending on the clinical signs in sows. For example, pyrexia is observed in 1-10% of sows, 1-2% of sows abort, returns to service are seen in 10-50% of cases, 7-35% of sows may deliver stillborn piglets, and 1-20% of sows farrow before 110 days of gestation (More et al. 2017). Interviews of veterinarians were used by de Paz et al. (2015) to estimate the animal-level prevalence of PRRS across the EU (Table E2). The total animal-level prevalence estimates of 36% for sows and 26% for weaner/finisher pigs for Denmark imply a within-herd prevalence of ~90% for sows and ~65% for weaner/finisher pigs based on the corresponding herd-level prevalence of 40% reported in 2015. The reported animal-level prevalence estimates for the UK, the Netherlands and Spain are substantially higher than those for Denmark, presumably reflecting higher herd-level prevalence in these countries. By extrapolating from the estimates provided for Denmark, it follows that the herd-level prevalence for Spain in particular is close to 100%. In contrast, Sweden is registered as free of PRRS (More et al. 2017).

Table D2. Point estimates of animal-level prevalence in 10 EU countries, after de Paz (2015). Countries of specific interest to this report are shown in bold.

	**Infected sows**		**Infected weaner/finisher pigs**	
	**with clinical signs**	**without clinical signs**	**with clinical signs**	**without clinical signs**
Germany	12 %	44 %	19 %	43 %
France	18 %	47 %	21 %	40 %
**Netherlands**	**14 %**	**59 %**	**21 %**	**43 %**
**Denmark**	**10 %**	**26 %**	**5 %**	**21 %**
**Spain**	**10 %**	**79 %**	**18 %**	**60 %**
Austria	17 %	27 %	23 %	17 %
**UK**	**13 %**	**39 %**	**16 %**	**31 %**
Italy	47 %	46 %	52 %	38 %
Belgium	22 %	69 %	31 %	57 %
Poland	13 %	41 %	18 %	39 %

Of the disease syndromes discussed in the previous section, the endemic syndrome of sows and weaner/finisher syndrome are likely to have the least impact on animal welfare. The weaner/finisher syndrome is associated with only mild clinical signs in a variable proportion of animals, and the syndrome is typically short-lived and without lasting consequence for the animals. The endemic syndrome of sows has an extremely high prevalence, but it is almost entirely limited to reproductive issues. Abortion, stillbirth and returns to service are not likely to be directly causative of physical discomfort in the sows, although the extent to which the behavioural and/or cognitive state of the animals may be affected is difficult to assess. The epidemic syndrome of sows and boars is associated with a greater welfare compromise to the affected animals, as well as a modest mortality rate, but is typically short-lived within a herd following initial introduction of the disease. The principle syndrome of concern to animal welfare is therefore likely to be the nursery syndrome. This syndrome is associated with a wide variety of clinical signs, many of which (e.g. anorexia, fever, diarrhoea, respiratory distress) may be associated with a substantial impact on welfare over a limited time period, and some of which (e.g. congenital abnormalities, poor growth rates, immunomodulation) may be associated with a relatively smaller impact that continues over the lifetime of the animal.

Given the high morbidity of the disease on affected farms, we therefore consider that PRRS may have a large impact on animal welfare. The recent reduction in herd-level prevalence in Denmark relative to the UK, Netherlands and in particular Spain has therefore most likely had a substantial positive impact on animal welfare in Denmark, and achieving the disease-free status of Sweden would further improve the situation. However, before affecting a control programme for PRRS it is also important to consider that the welfare implications of the epidemic syndrome of sows and boars may become relatively more important if the risk of re-introduction of PRRS onto a naïve farm is sufficiently high. It is therefore likely to be preferable from an animal welfare perspective to focus efforts on limiting inter-farm spread before enforcing eradication of the disease on farms that are at a high risk of reinfection. Fortunately, this goal is likely to be reasonably well aligned with the economic drivers of disease eradication.

D5. References

Antunes, A. C. L. *et al.* (2015) ‘Spatial analysis and temporal trends of porcine reproductive and respiratory syndrome in Denmark from 2007 to 2010 based on laboratory submission data’, *BMC Veterinary Research*. BMC Veterinary Research, 11(1), pp. 1–11. doi: 10.1186/s12917-015-0617-0.

Balasuriya, U. B. and Carossino, M. (2017) ‘Reproductive effects of arteriviruses: equine arteritis virus and porcine reproductive and respiratory syndrome virus infections’, *Current Opinion in Virology*. Elsevier B.V., 27, pp. 57–70. doi: 10.1016/j.coviro.2017.11.005.

Baron, T. *et al.* (1992) ‘Report on the first outbreaks of the porcine reproductive and respiratory syndrome (PRRS) in France. Diagnosis and viral isolation.’, *Annales de recherches vétérinaires. Annals of veterinary research*, 23(2), pp. 161–166.

Berton, P. *et al.* (2017) 'Evaluation of porcine reproductive and respiratory syndrome stabilization protocols in 23 French Farrow-to-finish farms located in a high-density swine area', *Porcine Health Management*. Porcine Health Management, 3, pp. 1–7. doi: 10.1186/s40813-017-0058-1.

Bilodeau, R. *et al.* (1994) 'Persistence of porcine reproductive and respiratory syndrome virus infection in a swine operation.', *Canadian journal of veterinary research = Revue canadienne de recherche vétérinaire*, 58(4), pp. 291–298.

Blaha, T. *et al.* (2000) 'The " colorful " epidemiology of PRRS', *Veterinary Research*, 31(1), pp. 77–83.

Dee, S. *et al.* (2018) 'A randomized controlled trial to evaluate performance of pigs raised in antibiotic-free or conventional production systems following challenge with porcine reproductive and respiratory syndrome virus', *PLoS ONE*, 13(12), pp. 1–15. doi: 10.1371/journal.pone.0208430.

Done, S. H. and Paton, D. J. (1995) 'Porcine reproductive and respiratory syndrome: clinical disease, pathology and immunosuppression', *The Veterinary Record*, 136(2), pp. 32–35.

Done, S. H., Paton, D. J. and White, M. E. G. (1996) 'Porcine reproductive and respiratory syndrome (PRRS): A review, with emphasis on pathological, virological and diagnostic aspects', *British Veterinary Journal*, 152(2), pp. 153–174. doi: 10.1016/S0007-1935(96)80071-6.

Frössling, J. *et al.* (2009) 'Probability of freedom from disease after the first detection and eradication of PRRS in Sweden: Scenario-tree modelling of the surveillance system', *Preventive Veterinary Medicine*, 91(2–4), pp. 137–145. doi: 10.1016/j.prevetmed.2009.05.012.

Goyal, S. M. (1993) 'Porcine reproductive and respiratory syndrome virus', *Theriogenology*, 66(3 SPEC. ISS.), pp. 655–662. doi: 10.1016/j.theriogenology.2006.04.024.

Hopper, S. A., White, M. E. C. and Twiddy, N. (1992) 'An outbreak of blue-eared pig disease (porcine reproductive and respiratory syndrome) in four pig herds in Great Britain', *The Veterinary Record*. The Veterinary Record, 131(7), pp. 140–144.

Jeong, J. *et al.* (2018) 'A comparison of the severity of reproductive failure between single and dual infection with porcine reproductive and respiratory syndrome virus (PRRSV)-1 and PRRSV-2 in late-term pregnancy gilts', *Transboundary and Emerging Diseases*, 65(6), pp. 1641–1647. doi: 10.1111/tbed.12921.

Kvisgaard, L. K. *et al.* (2017) 'Genetic and biological characterization of a Porcine Reproductive and Respiratory Syndrome Virus 2 (PRRSV-2) causing significant clinical disease in the field', *Veterinary Microbiology*. Elsevier, 211(October), pp. 74–83. doi: 10.1016/j.vetmic.2017.10.001.

Lewis, C. R. G. *et al.* (2007) 'Genetic perspectives on host responses to porcine reproductive and respiratory syndrome (PRRS)', *Viral Immunology*, 20(3), pp. 343–357. doi: 10.1089/vim.2007.0024.

Lunney, J. K., Benfield, D. A. and Rowland, R. R. R. (2010) 'Porcine reproductive and respiratory syndrome virus: An update on an emerging and re-emerging viral disease of swine', *Virus*

Research, 154(1–2), pp. 1–6. doi: 10.1016/j.virusres.2010.10.009.

Mengeling, W. L., Lager, K. M. and Vorwald, A. C. (2000) ‘The effect of porcine parvovirus and porcine reproductive and respiratory syndrome virus on porcine reproductive performance’, *Animal Reproduction Science*, 60–61, pp. 199–210. doi: 10.1016/S0378-4320(00)00135-4.

More, S. *et al.* (2017) ‘Assessment of listing and categorisation of animal diseases within the framework of the Animal Health Law (Regulation (EU) No 2016/429): porcine reproductive and respiratory syndrome (PRRS)’, *EFSA Journal*, 15(7). doi: 10.2903/j.efsa.2017.4949.

Nathues, C. *et al.* (2016) ‘An Outbreak of Porcine Reproductive and Respiratory Syndrome Virus in Switzerland Following Import of Boar Semen’, *Transboundary and Emerging Diseases*, 63(2), pp. e251–e261. doi: 10.1111/tbed.12262.

Nathues, H. *et al.* (2017) ‘Cost of porcine reproductive and respiratory syndrome virus at individual farm level – An economic disease model’, *Preventive Veterinary Medicine*. Elsevier B.V., 142, pp. 16–29. doi: 10.1016/j.prevetmed.2017.04.006.

Nodelijk, G. (2002) ‘Porcine reproductive and respiratory syndrome (prrs) with special reference to clinical aspects and diagnosis: A review’, *Veterinary Quarterly*, 24(2), pp. 95–100. doi: 10.1080/01652176.2002.9695128.

Nodelijk, G. *et al.* (2003) ‘A review of porcine reproductive and respiratory syndrome virus in Dutch breeding herds: Population dynamics and clinical relevance’, *Preventive Veterinary Medicine*, 60(1), pp. 37–52. doi: 10.1016/S0167-5877(03)00081-3.

de Paz, X. *et al.* (2015) ‘PRRS prevalence in Europe: Perception of the pig veterinary practitioners - PRRS.com’, *Esphm 2015*, 24(2), p. 1. Available at: https://www.prrs.com/en/publications/abstracts/prrs-prevalence-europe-perception-the-pig-veterinary-practitioners/.

Pejsak, Z. and Markowska-Daniel, I. (1997) ‘Losses due to porcine reproductive and respiratory syndrome in a large swine farm’, *Comparative Immunology, Microbiology and Infectious Diseases*, 20(4), pp. 345–352. doi: 10.1016/S0147-9571(97)00010-6.

Le Potier, M. F. *et al.* (1997) ‘Results of a control programme for the porcine reproductive and respiratory syndrome in the French “Pays de la Loire” region’, *Veterinary Microbiology*, 55(1–4), pp. 355–360. doi: 10.1016/S0378-1135(96)01318-1.

Prieto, C. and Castro, J. M. (2005) ‘Porcine reproductive and respiratory syndrome virus infection in the boar: A review’, *Theriogenology*, 63(1), pp. 1–16. doi: 10.1016/j.theriogenology.2004.03.018.

Reeth, K. Van (1997) ‘Pathogenesis and clinical aspects of a respiratory porcine reproductive and respiratory syndrome virus infection’, *Veterinary Microbiology*, 55, pp. 223–230.

Rossow, K. D. (1998) ‘Porcine Reproductive and Respiratory Syndrome’, *Veterinary Pathology*,

35(1), pp. 1–20.

Sinn, L. J. *et al.* (2016) 'Emergence of a virulent porcine reproductive and respiratory syndrome virus (PRRSV) 1 strain in Lower Austria', *Porcine Health Management*. Porcine Health Management, 2, pp. 1–10. doi: 10.1186/s40813-016-0044-z.

Toft, N. and Veterinærinstituttet, D. T. U. (2015) 'ØKONOMISK BEREGNING AF OMKOSTNINGEN VED EN NATIONAL PRRS- Sammendrag Baggrund', (1032), pp. 1–17.

Wensvoort, G. (1993) 'Lelystad virus and the porcine epidemic abortion and respiratory syndrome', *Veterinary Research*, 24(2), pp. 117–124.

Young, B. *et al.* (2010) 'Clinical signs and their association with herd demographics and porcine reproductive and respiratory syndrome (PRRS) control strategies in PRRS PCR-positive swine herds in Ontario', *Canadian Journal of Veterinary Research*, 74(3), pp. 170–177.
